# Atypical leishmaniasis: A global perspective with emphasis on the Indian subcontinent

**DOI:** 10.1371/journal.pntd.0006659

**Published:** 2018-09-27

**Authors:** Lovlesh Thakur, Kiran K. Singh, Vinay Shanker, Ajeet Negi, Aklank Jain, Greg Matlashewski, Manju Jain

**Affiliations:** 1 Department of Animal Sciences, Central University of Punjab, Bathinda, Punjab, India; 2 Department of Geography and Geology, Central University of Punjab, Bathinda, Punjab, India; 3 Department of Dermatology and Venereology, Maharishi Markandeshwar Medical College and Hospital, Sultanpur, Kumarhatti, Solan, Himachal Pradesh, India; 4 Department of Dermatology, Indira Gandhi Medical Centre, Shimla, Himachal Pradesh, India; 5 Department of Microbiology and Immunology, McGill University, Montreal, Quebec, Canada; 6 Department of Biochemistry and Microbial Sciences, Central University of Punjab, Bathinda, Punjab, India; U.S. Food and Drug Administration and Center for Biologics Evaluation and Research, UNITED STATES

## Abstract

**Background:**

Among the neglected tropical diseases, leishmaniasis continues to be prevalent in many tropical and subtropical countries despite international, national, and local efforts towards its control and elimination over the last decade. This warrants a critical evaluation of such factors as under-reporting, asymptomatic infections, post kala azar dermal leishmaniasis (PKDL) cases, and drug resistance. In this review, we highlight lesser-understood atypical presentations of the disease involving atypical parasite strains against a background of classical leishmaniasis with a focus on the Indian subcontinent.

**Methods and findings:**

A literature review based on endemic areas, the nature of disease manifestation, and underlying causative parasite was performed with data collected from WHO reports for each country. Searches on PubMed included the term ‘‘leishmaniasis” and “leishmaniasis epidemiology” alone and in combination with each of the endemic countries, *Leishmania* species, cutaneous, visceral, endemic, non-endemic, typical, classical, atypical, and unusual with no date limit and published in English up to September 2017. Our findings portray a scenario with a wider distribution of the disease in new endemic foci, with new discoveries of parasite-driven atypical disease manifestations in different regions of the world. Unlike the classical picture, some *Leishmania* species are associated with more than one disease presentation, e.g., the *L*. *donovani* complex, generally associated with the visceral form, is now also associated with a cutaneous disease presentation, while *L*. *tropica* species complex, known to cause cutaneous disease, can cause viscerotropic disease. This phenomenon points towards the discovery of novel parasite variants as etiologic agents of atypical disease manifestations and represents an excellent opportunity to identify and study genes that control disease virulence and tropism.

**Conclusions:**

The increased recognition of atypical leishmaniasis as an outcome of parasite variants has major implications for leishmaniasis control and elimination. Identifying molecular correlates of parasite isolates from distinct regions associated with different disease phenotypes is required to understand the current epidemiology of leishmaniasis in regions with atypical disease.

## Introduction

Leishmaniasis is one of the most neglected infectious tropical diseases and is caused by an intracellular protozoan belonging to the genus *Leishmania*. In essence, it is a disease complex exhibiting a gradation of clinical manifestations ranging from cutaneous leishmaniasis (CL) with skin lesions to mucocutaneous leishmaniasis (MCL) involving the mucous membrane to a lethal systemic form, visceral leishmaniasis (VL). The disease has a wide geographical occurrence, covering 97 countries and territories with endemic foci for each of the different clinical forms [1,2]. More than 20 *Leishmania* species are known to circulate in endemic foci in Africa, Asia, the Middle East, the Mediterranean region, Central-South America, and southern Europe. The *L*. *donovani* and *L*. *infantum/chagasi* complex is responsible for VL; the *L*. *major*, *L*. *tropica*, *L*. *aethiopica*, and *L*. *mexicana* complex causes CL, and the subgenus *L*. *Viannia* complex causes CL and MCL as per the classical association of specific parasite species with distinct clinical outcomes [3].

More recently, leishmaniasis as a disease is breaking out of its classical boundaries and is reported from new geographic locations with unusual atypical disease manifestations with novel parasite variants. This review highlights the atypical disease epidemiology wherein variants of *Leishmania* species classically associated with distinct clinical phenotypes can also cause atypical disease. Hence, atypical disease manifestations caused by atypical parasite isolates in comparison with classical clinical phenotypes caused by typical parasite species are discussed. An attempt to correlate genetically distinct parasite isolates with the atypical disease phenotype from different geographical regions is presented to identify genotypes with specific phenotypes [4–9]. In lieu of such parasite-driven atypical disease manifestations, newer challenges to diagnose and treat the disease are foreseen.

**Fig 1:**
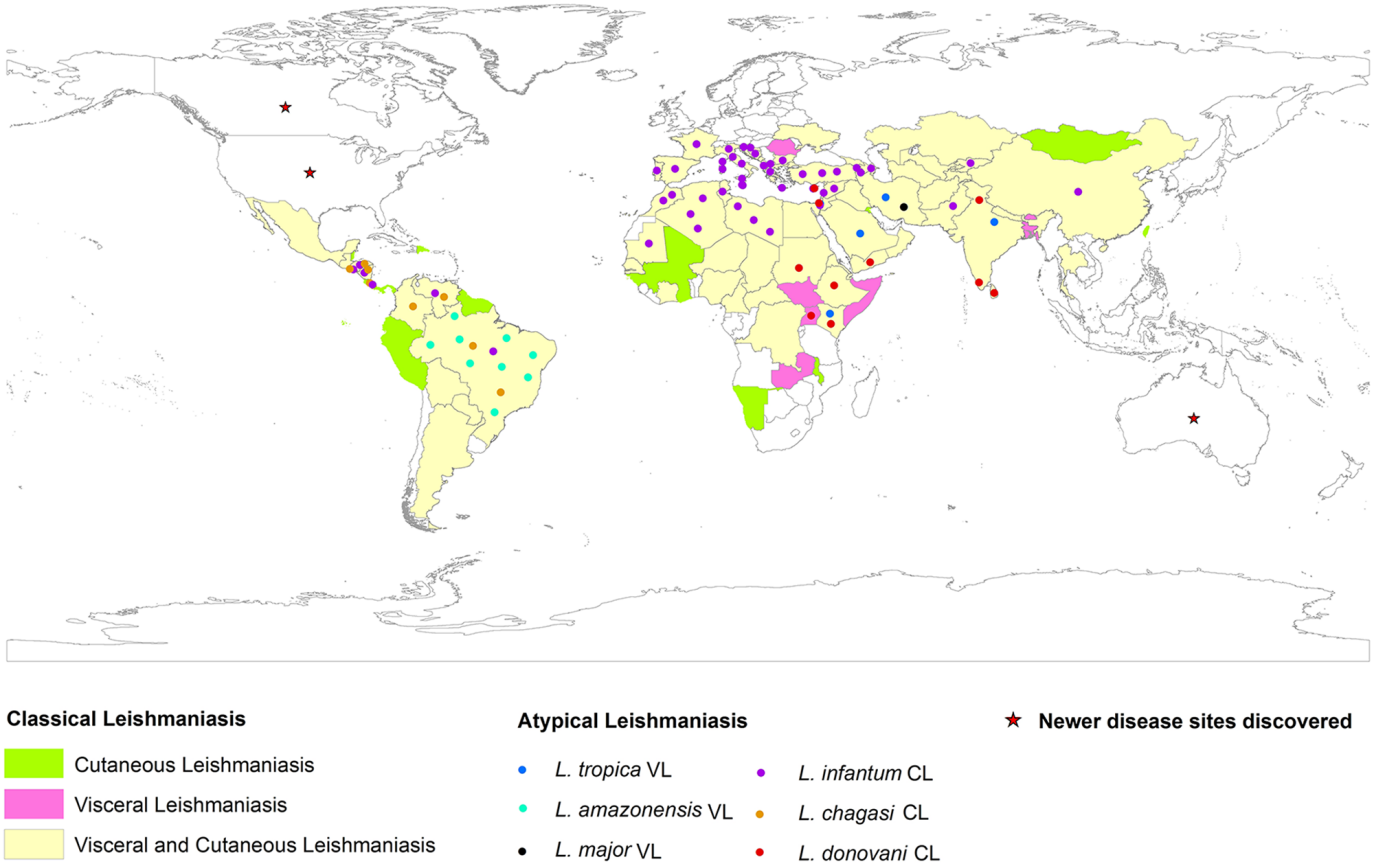
Global distribution of classical and atypical leishmaniasis in New and Old World countries. Solid rectangles indicate region-specific prevalence of classical disease type; CL (green rectangle), VL (pink rectangle), VL and CL (yellow rectangle). Regions with solid circles indicate atypical CL and VL disease form. Different color code of solid circles discriminate the causative parasite species responsible for atypical leishmaniasis; *L*. *tropica* VL (blue solid circles), *L*. *amazonensis* VL (aquamarine solid circles), *L*. *major* VL (black solid circles), *L*. *infantum* CL (purple solid circles), *L*. *chagasi* CL (gold solid circles), and *L*. *donovani* CL (red solid circles). Regions with red stars represent newer disease sites discovered. The map was created using ArcGIS 10.3. Regional distributions of disease were georeferenced with UTM projection taking WGS84 datum. CL, cutaneous leishmaniasis; VL, visceral leishmaniasis.

**Fig 2:**
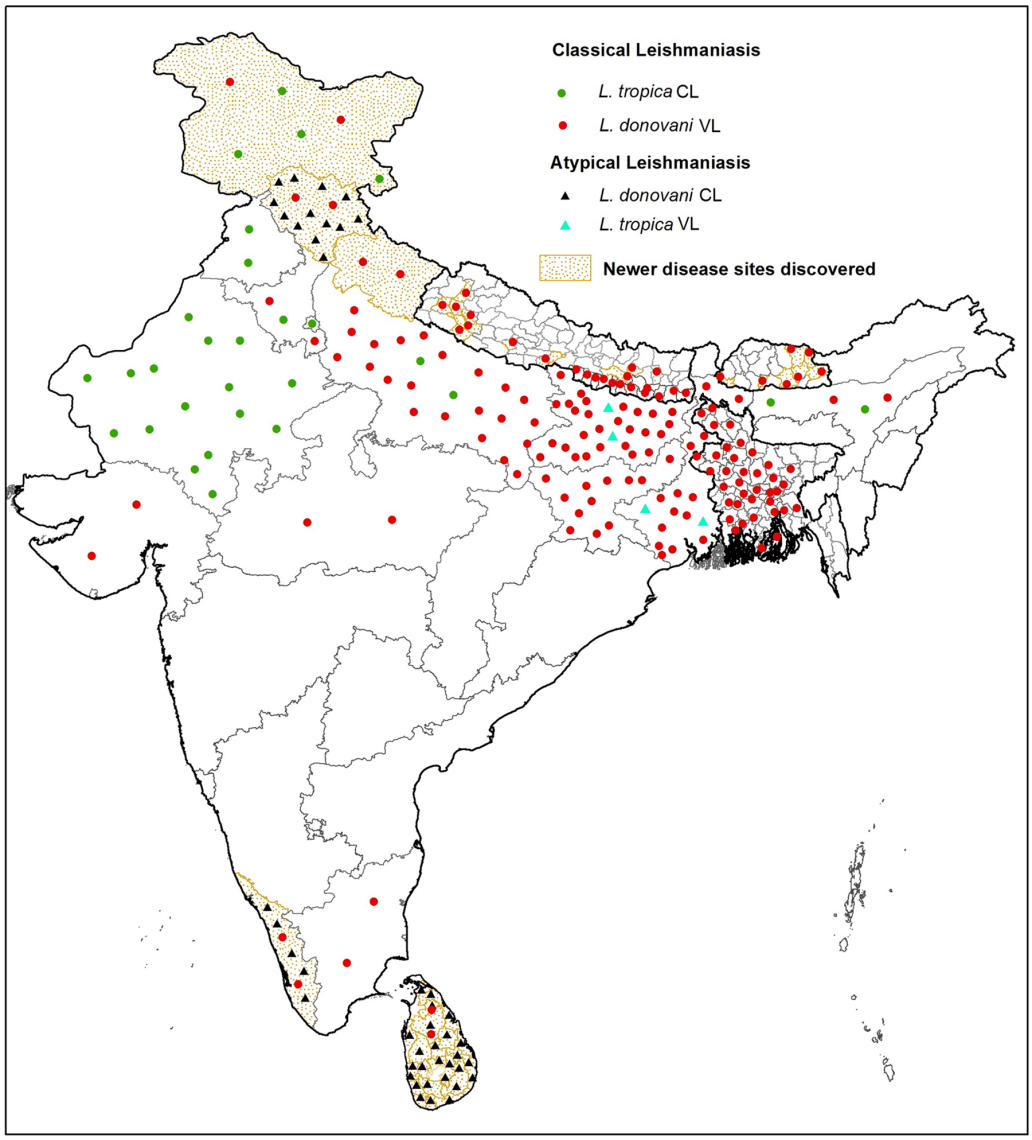
Geographical distribution of classical and atypical leishmaniasis cases in Indian subcontinent. Solid circles represent classical CL and VL leishmaniasis, and solid triangles represent atypical CL and VL leishmaniasis with different color codes. Green solid circles and red solid circles represent classical *L*. *tropica* CL and *L*. *donovani* VL, respectively. Aquamarine solid triangles and black solid triangles represent atypical *L*. *tropica* VL and *L*. *donovani* CL, respectively. Regions with dotted gold background indicate newer disease sites discovered. Solid colored circles and triangles indicate prevalence of different disease forms and do not represent the number of leishmaniasis cases. The map was created using ArcGIS 10.3. Regional distributions of disease were georeferenced with UTM projection taking WGS84 datum. CL, cutaneous leishmaniasis; VL, visceral leishmaniasis.

The association between the infecting *Leishmania* species and the clinical outcome has been one of the most intriguing areas of leishmaniasis research. While *L*. *donovani* complex infect visceral organs, CL-causing *L*. *tropica/L*. *major* complex remain in the skin, showing different degrees of parasite dissemination. Historically, these clinical-parasitological features were the basis of differentiating the infecting parasite species. More recently, the significance of parasite determinants in driving biology of disease outcome is being explored, with demonstration of novel parasite genotypes in relation to distinct disease phenotypes [10,11]. Thus, the existence and circulation of parasite variants can be the basis of atypical disease manifestations, and the study of these variants can help to identify genes associated with differential tropism and virulence. Multiple incidences that highlight newer and lesser understood aspects of such parasite-mediated atypical disease forms are reviewed to illustrate the current disease epidemiology at the global level, with a focus on the Indian subcontinent (Figs 1 and 2, S1 and S2 Tables).

## Visceral leishmaniasis

VL is the systemic form of the disease that can be fatal if not treated. An estimated 50,000 to 90,000 new cases occur every year, with almost all leishmaniasis fatalities coming from this form of the disease [2]. More than 90% of the VL cases occur in only a few countries, including India, Brazil, Ethiopia, Somalia, Kenya, South Sudan, and Sudan [2] (Fig 1 and S1 Table). Asymptomatic visceral infections along with disease sequelae in the form of post kala azar dermal leishmaniasis (PKDL) represent additional complexities of the disease. There is a trend towards wider geographical dissemination of VL in newer endemic areas [12–17]. Moreover, there are increased reports on atypical VL cases wherein parasite species classically associated with cutaneous disease cause the viscerotropic phenotype (Fig 1 and S1 Table).

### Atypical visceral leishmaniasis

A series of reports imply that *L*. *tropica*, *L*. *amazonensis*, and *L*. *major*, classically associated with CL, can rarely cause VL disease.

### *L*. *tropica* visceral leishmaniasis

Although *L*. *tropica* is typically associated with CL in the Old World, *L*. *tropica* isolates that exhibit viscerotropic characteristics have been reported from patients with VL [18–23]. Such cases have been reported from different regions, including Saudi Arabia, Iran, Kenya, Israel, and India. Some United States veterans from Operation Desert Storm returning from Bahrain and Saudi Arabia were diagnosed with systemic infection with *L*. *tropica* resulting in a milder visceral disease phenotype than classic VL [20]. Northwestern and southern Iran are foci for VL, with *L*. *infantum* the dominant parasite species; however, out of 64 VL cases, one was reported to be caused by *L*. *tropica* [19]. Similar *L*. *tropica*–associated VL cases, accompanied by diffuse cutaneous leishmaniasis (DCL), have been reported from southern Iran [18]. In Kenya, both cutaneous and visceral forms are prevalent in their classical format. However, instances of atypical visceral manifestation caused by *L*. *tropica* variants have also been reported from Kenya, and these patients showed unresponsiveness to sodium-stibogluconate treatment [21]. VL is relatively rare in adults in Israel, and most cases have been in children from western Galilee and around Bethlehem [24]. CL is endemic in southern Jordan Valley and Negev regions, with *L*. *major*/*L*. *tropica* as the causative parasites [25]. With this classical scenario, an atypical VL case has been reported with the parasite isolate possessing features specific to both *L tropica* and *L*. *donovani* [22]. In India, VL is the prominent disease form caused by *L*. *donovani*, with fewer incidences of CL caused by *L*. *tropica*. However, in northeast India, a fraction of parasite isolates from classical VL cases have been typed as *L*. *tropica* [4].

### *L*. *amazonensis* visceral leishmaniasis

*L*. *amazonensis* generally associated with CL is reported to cause all three forms of leishmaniasis in humans, including CL, MCL, and VL along with PKDL in Bahia, Brazil [26]. Additionally, *L*. *amazonensis* isolates from patients with different clinical manifestations exhibited genetic heterogeneity in a study carried out in northeastern Brazil [27].

### *L*. *major* visceral leishmaniasis

*L*. *major* is classically associated with CL; however, more recently, viscerotropic dissemination of *L*. *major* has been evaluated in southwestern Iran. The region is endemic for VL caused by *L*. *infantum* and CL caused by *L*. *major*. Molecular diagnosis revealed *L*. *major* as the agent in a VL case in the Bushehr province of Iran. The patient initially exhibited chronic cutaneous disease refractory to treatment with subsequent visceral dissemination [28].

## Cutaneous leishmaniasis

CL is the most common manifestation of the disease, with approximately 0.6 to 1 million new cases every year. With prevalence in the Americas, the Mediterranean basin, the Middle East, and Central Asia, approximately two-thirds of the cases are concentrated in Afghanistan, Algeria, Brazil, Colombia, Iran, and the Syrian Arab Republic [2]. CL is a highly neglected condition because it is generally self-healing and rarely fatal. *L*. *tropica/L*. *major* and *L*. *aethiopica* are the predominant etiologic agents in the Old World, whereas *L*. *mexicana* and *L*. *(V*.*) braziliensis* complex are predominant in the New World. *L*. *tropica* has both anthroponotic and zoonotic transmission, whereas others are only zoonotic. Discovery of atypical CL cases in newer endemic areas as well in places where the disease is present in one of its classical forms is evident. Such atypical CL cases are associated with discrete parasite variants belonging to parasite species that generally cause VL (Fig 1 and S1 Table).

### Atypical cutaneous leishmaniasis

Multiple reports from known and newer leishmaniasis-inflicted regions have implicated *L*. *donovani*, *L*. *infantum*/*L*. *chagasi* as the causative agent of CL, at times with mucosal manifestations.

### *L*. *infantum/L*. *chagasi* cutaneous leishmaniasis

*L*. *infantum* is known predominantly as the etiological agent of VL in the Mediterranean region compared to its lesser-understood role as a causative agent of CL. The atypical *L*. *infantum* CL has been reported in parts of North Africa and Europe, as well as the Americas [29,30] (S1 Table). Historically, it was initially identified in CL patients from Pyrenees-Orientalis [31]. With considerable polymorphism, *L*. *infantum* is known to cause sporadic CL cases in areas endemic for VL in the Mediterranean basin [30]. In northern Africa, CL is predominately caused by *L*. *tropica* and *L*. *major* over the atypical CL caused by *L*. *infantum*. Specific countries in the region with CL caused by *L*. *infantum* include Algeria, Morocco, and Tunisia [29] (S1 Table). *Leishmania* isolates from CL cases in Lebanon and Syria have also been characterized as *L*. *infantum* variants [32]. In southern and western Europe, *L*. *infantum* is the only species responsible for CL cases, with reports of autochthonous CL from Spain, Italy, Malta, Cyprus, Greece, France, and Portugal [16,30] (S1 Table). Atypical CL caused by *L*. *infantum* is also reported in the New World in areas where CL is also caused by *L*. *mexicana* and *L*. *braziliensis*. *L*. *infantum/chagasi* CL cases have been reported from Brazil, Venezuela, Honduras, El Salvador, Nicaragua, Costa Rica, and Colombia, such that the two organisms are considered synonymous based on molecular analysis of multiple *L*. *infantum*/*chagasi* strains of different origin [33] (S1 Table). Noticeably, the *L*. *infantum/chagasi* atypical CL cases documented in different regions are predominantly autochthonous in nature and mostly represent immunocompetent hosts, implying a critical role of parasite determinants in disease outcome.

### *L*. *donovani* cutaneous ceishmaniasis

*L*. *donovani* is the species responsible for VL in the Old World, with endemic foci in northeastern India, Bangladesh, Nepal, Bhutan, and China in Asia and Sudan, Ethiopia, Kenya, Somalia, Uganda, and Eritrea in Africa. However, some of these areas and newer regions are also reporting autochthonous atypical CL caused by *L*. *donovani*. In Sudan, VL is caused by *L*. *donovani* MON-82 zymodeme, whereas CL, also endemic in Sudan, is associated with the *L*. *major* MON-74 zymodeme. However, parasite isolates from cutaneous ulcers from CL patients presented at Khartoum were identified as *L*. *donovani* MON-82 [34]. Similarly, atypical CL cases have been reported to be caused by *L*. *donovani* zymodeme Z6 in Kenya along with the classical CL caused by *L*. *major*, *L*. *tropica*, and *L*. *aethiopica* [35]. In northwest Yemen, the majority of CL cases are caused by *L*. *tropica* along with occasional sympatric occurrence by *L*. *donovani* complex [36]. Further variation of the CL disease caused by *L*. *donovani* exists wherein the parasite is detected in blood culture with no visceral manifestation [37].

Consistent with reports of atypical VL and CL cases due to atypical *Leishmania* variants at the species/subspecies level, it is apparent that manifestation of leishmaniasis is mainly determined by the parasite’s ability to cause different clinical outcomes. Apart from prevalence of atypical leishmaniasis discussed above, leishmaniasis is increasingly present in newer regions previously known to be free of the disease. Multiple reports on exotic disease presence in Canada, the US, and Northern Europe also exist wherein patients reveal a history of travel to one of the regions endemic for the disease [13,16,38].

## Indian subcontinent

The Indian subcontinent comprises regions endemic for leishmaniasis, including India, Bangladesh, Nepal, and more recently, Bhutan and Sri Lanka. Newer and expanding endemic sites and parasite species/variants causing atypical clinical manifestations are also reported from this part of the world resulting in newer challenges in achieving elimination targets (Fig 2 and S2 Table).

### Visceral leishmaniasis in the Indian subcontinent

VL, locally known as kala azar, is a major public health problem, with more than half the worldwide disease burden coming from the Indian subcontinent. With *L*. *donovani* as the known causative organism, most VL cases have been reported in northeastern India, western and central districts of Bangladesh, and the southeastern Terai region of Nepal, with more recent incidences in Bhutan and Sri Lanka (Fig 2 and S2 Table). In lieu of high disease burden, India, Bangladesh, and Nepal initiated programs in 2005 to eliminate VL with the target of less than one case per 10,000 in endemic areas by 2015 [39]. This target has largely been met in Bangladesh and Nepal; however, in India, the target has now been extended to 2019. In Nepal, 12 districts known to be endemic for VL are reported to have achieved the elimination target, with a reduction in total number of cases from 1,463 in 2005 to 150 in 2016 [1,40–42]. However, new VL case reports from previously non-endemic eastern and western hilly regions of Nepal are a rising concern [43,44]. In Bangladesh, all 100 upazilas reported to be endemic for VL have achieved the elimination target, and the number of VL cases has declined from 6,892 cases in 2005 to 255 in 2016 [1,40,41,45]. There is, however, a rising incidence of PKDL in VL-endemic areas of Bangladesh [42,46]. Bhutan is a more recent geographical extension with a series of sporadic VL cases since 2005 [42,47]. In Sri Lanka, VL incidence reports are more recent, with the first autochthonous case detected in 2007 from the Anuradhapura district, and the number of cases are few but increasing [17,48].

In India, visceral disease is concentrated in eastern states of the country, including Bihar, West Bengal, Uttar Pradesh, and Jharkhand [3,42]. Bihar is the most affected state with 90% of the total caseload, although the VL elimination target has been achieved in 366 out of 456 blocks in Bihar [40,41]. Total VL cases reported in India have been reduced from 32,803 in 2005 to 6,231 in 2016 [1]. Apart from the aforementioned states with high caseloads, reports on resurgence of disease in the states of Gujarat, Assam, Tamil Nadu along with the discovery of newer afflicted regions have been reported during the last decade [12,49,50]. Many of the newer foci are reported to have indigenous disease cases, whereas some are reported to result from migratory populations. Sporadic VL cases have been reported from Kerala, Morena, and Malwa regions of Madhya Pradesh and Haryana [12,51–54]. Case reports of the disease from sub-Himalayan parts, including Uttarakhand, Himachal Pradesh, Jammu, and Kashmir, are of special interest because VL is typically a disease of low altitude [14,15,55–57]. In fact, leishmaniasis is reported as the most common cause of bone marrow infections in the North Himalayan region [58]. In this context, the disease along with the vector has been confirmed from previously non-endemic hilly regions—Kumaon and Garhwal of Uttarakhand—with reports of a gradual increase in incidence of indigenous cases such that the state is now considered a new focus of VL [55,59,60]. Another hilly state considered as a new pocket of VL is Himachal Pradesh, with reports on increased incidence of VL cases from 2004 onwards [56]. Pockets discovered for the disease are on the rise in the southeastern area along the Sutluj river belt comprising Kinnaur, Shimla, and Kullu districts along with northwestern Chamba region in the Ravi river belt of Himachal Pradesh [15,57]. Dominated by a classical visceral presentation, the cases are reported to be indigenous with a local vector transmission [61]. VL has been rarely reported from Jammu and Kashmir, although there is a case report of VL in a native Kashmiri boy in 2009 and a recent autochthonous case of a young male from Bani, in the district Kathua, Jammu [62,63]. Sporadic VL cases are also reported from the Basholi area in Jammu and Kashmir along the Ravi river valley [15]. Although there is an overall decline in VL incidence in India due to the VL elimination program in the highly endemic areas, the newer cases from typically non-endemic regions appear to be increasing, and this is of critical importance to avoid resurgence and to achieve sustainable elimination of the disease from the country.

### Atypical visceral leishmaniasis in the Indian subcontinent

Apart from the newer VL foci discovered, a series of atypical disease manifestations consistent with global trends is also present in India. With *L*. *donovani* as the known causative agent for VL in India, a few clinical isolates from northeastern India have been reported to be *L*. *tropica* based on species-specific PCR-restriction fragment length polymorphism (PCR-RFLP) [4]. A case of VL caused by *L*. *tropica* was also reported from Himachal Pradesh [5]. Furthermore, strains of *L tropica* isolated from Indian VL patients exhibit genetic dissimilarities from those isolated from CL patients. The differences are thought to be the possible basis of altered tropism and pathogenicity of *L*. *tropica* variants causing VL or CL manifestations [5].

### Cutaneous leishmaniasis in the Indian subcontinent

Cutaneous manifestation is also prevalent in specific regions of the Indian subcontinent where CL is historically endemic in the dry and hot area of the Thar Desert in Rajasthan, with Bikaner as the major focus of the disease [64]. Molecular characterization revealed *L*. *tropica* as the infecting parasite [12,64]. Sporadic CL cases are also reported from Punjab, Assam, Haryana, Delhi, and Varanasi [12]. A high number of CL patients have been reported from Doda, Kishtwar, and Poonch districts in Jammu and two hilly areas, Uri and Karnah, from the Kashmir Valley [65,66]. In Nepal, sporadic CL cases have been referred, with *L*. *major* as the causative agent in one such case [67]. More recently, case reports of CL in immigrants from the Middle East have been documented in Bangladesh [68].

### Atypical cutaneous leishmaniasis in the Indian subcontinent

CL is now reported from newer endemic regions in Sri Lanka and India in the form of atypical CL caused by atypical *L*. *donovani* variants. Patients exhibiting cutaneous lesions with no symptoms of VL are being reported with a high incidence rate in Sri Lanka such that the disease is now considered endemic in the country. CL in Sri Lanka was initially considered a traveler’s disease until autochthonous cases were detected from 1992 onwards, and since then, the number of such cases have been increasing [69]. Skin lesion isolates have been molecularly identified as *L*. *donovani* MON-37 showing close genotypic relation as well as distinct molecular differences when compared with the *L*. *donovani* MON-2 zymodeme, which causes VL in India [6]. In India, an increased frequency of CL cases from Kerala and Himachal Pradesh are being reported, with specific pockets in these states now considered endemic for the disease. To begin with, no CL case had been documented in Kerala before 1988 until two imported cases were reported in Thiruvananthapuram, followed by reports of indigenous cases from Malappuram and Kollam. Since then, a number of cases have been documented in specific forest settlements of the Kani tribe and tribal villages in the Agasthyamala Biosphere Reserve forest along the Western Ghats, Kerala [9,70]. Based on the number of cases detected, this region is now considered endemic for atypical CL. The disease form is atypical, as the clinical isolates have been identified as *L*. *donovani* variants, similar to those identified in Sri Lanka from atypical CL cases. Detection of *P*. *argentipes*, naturally infected with *L*. *donovani*, further endorses the area to be an autochthonous focus of atypical CL [9,71]. Another state witnessing atypical CL is Himachal, with its own peculiar characteristics described below.

### Himachal Pradesh, India: A new endemic focus of atypical leishmaniasis

Himachal Pradesh, a previously non-endemic area, is emerging as a new disease state in Northwest India. Based on documentation of the local transmission vector, demonstration of circulating anti-rK39 antibody in patients as well as the canine population and a series of case reports, the region is now considered an endemic focus, with a peculiar coexistence of cutaneous and visceral forms of the disease [7,15,61,72]. The frequency of CL over VL cases is much higher, although both forms are coendemic. The VL zone includes the southeastern area along the Sutluj river belt comprising the Kinnaur, Shimla, and Kullu districts, with recent case reports from northwestern Chamba region along the Ravi river belt [15]. Visceral disease resembles the classical presentation, with *L*. *donovani* the underlying causative organism. *L*. *donovani* in the region is also responsible for dermotropic manifestation, with the first ever case studies of CL reported from the Kinnaur district spreading further to the bordering regions of Shimla district (Rampur and Kumarseain) and Kullu district (Nirmand) along the Satluj river [73]. Molecular characterization of the infecting parasite from a cohort of CL and VL patients revealed atypical *L*. *donovani* as the major cause of CL along with *L*. *tropica* [7]. Thus, the Satluj river belt is, at present, an established coendemic zone for VL and CL manifestation probably caused by viscerotropic and dermotropic variants of *L*. *donovani* complex. It is noteworthy that molecular analysis of parasite isolates, implying *L*. *donovani* as the predominant pathogen causing CL with fewer CL cases caused by *L*. *tropica*, is considered preliminary since molecular analysis was performed on only a few samples [7]. Whole-genome sequencing of clinical isolates from the region is required to establish phylogenetic origin and genetic relatedness of the parasite species/subspecies circulating in the region. In this regard, our laboratory has taken up molecular characterization of approximately 60 clinical isolates from this region. Our results corroborate the initial finding of *L*. *donovani* being the major CL causing species. Delineating specific genetic differences, based on distinct gene sequences and possible polymorphisms defining parasite variants in this region compared to standard genotypes indigenous to the Indian subcontinent is underway. So far, our work points toward the existence of novel *L*. *donovani* variants in the region as the possible explanation for the atypical CL. Several cases of mucocutaneous manifestation are also reported from the region [74]. The heterogeneous disease manifestation with prevalence of cutaneous, mucocutaneous, and visceral phenotype requires a comprehensive study to understand disease etiology in terms of characterizing parasite variants and immune heterogeneity of patients to understand the unique disease presentation in the region. This is required to help ensure this region does not become a new area for a major outbreak of VL.

## Genetic basis: Disease outcome: A confederation of parasite and host biology

The gradation of clinical variations seen in leishmaniasis is explained in terms of the distinct parasite species involved and the integrity of the host defense system. Among these, the specific parasite species is considered the dominating determinant for differential disease outcomes. Ongoing research is underway to determine the molecular mechanism of differential tissue tropism and pathogenic capabilities of parasite species/subspecies causing visceral, cutaneous, and mucocutaneous disease. The genetic analysis of atypical parasite isolates can help explain the biology of atypical disease manifestations. Multilocus microsatellite typing (MLMT)-based comparison of *L*. *tropica* isolates from classical CL cases in Bikaner to *L*. *tropica* isolates from atypical VL cases in Bihar exhibited genetic differences potentially correlated with the different disease phenotypes [5]. A recent study based on multiple PCR-RFLP and MLMT markers demonstrated significant genetic polymorphism in VL-causing *L*. *donovani* isolates, belonging to the most common Indian zymodeme, MON-2 from northeast India [75]. Similarly, characterization of parasite isolates as typical and atypical variants from different endemic sites has been performed using isoenzyme electrophoretic mobility, PCR-RFLP, and sequence analysis of specific gene/intergenic regions [4,6,7,9,32,34,48]. More recently, whole-genome sequencing of different parasite species, patient-derived clinical isolates, and laboratory-manipulated parasite variants is providing novel insight into the role of parasite genes in determining drug resistance, PKDL phenotype, and atypical disease manifestation [8,76–79]. The mechanism of differential tissue tropism of parasite species and atypical parasite variants is now becoming better understood in terms of specific parasite genes, gene polymorphisms, and gene amplification and/or deletion in relation to distinct disease phenotypes. A relatively small number of genes have been shown to be differentially present in viscerotropic species compared to dermotropic species, and many of these genes have no identified functional annotation. Based on studies employing gene transfer of *L*. *donovani* genes into *L*. *major* wherein the transgenic parasites survived better in the viscera of infected mice, some of the *L*. *donovani*–specific genes shown to support viscerotropic infection include the A2 gene cluster, Ld28.0340, a cytosolic protein of unknown function, Ld15.0900, a nucleotide sugar transporter, and Ld36.2480, a glyceraldehyde-3-phosphate dehydrogenase [10,11]. The gene-specific protein products confer better survival of intracellular amastigotes at higher temperature and oxidative stress encountered in visceral tissue.

With a limited number of species-specific genes, gene copy number variations and single-nucleotide polymorphisms appear to be important in determining genetic variability and differential tissue tropism of parasite variants leading to distinct disease phenotypes. In this regard, whole-genome comparison of VL-causing isolates and atypical CL-causing isolates of *L*. *donovani* variants belonging to the same MON-37 zymodeme from Sri Lanka is particularly interesting. A decreased copy number of the virulence A2 genes and a mutation in the Rag C GTPase have been identified as determinants for the attenuation of the CL-causing *L*. *donovani* strain in Sri Lanka [8]. Proteome comparison of the *L*. *donovani* cutaneous and visceral disease isolates from Sri Lanka showed differential expression of proteins related to translation, biosynthetic processes, and antioxidant protection, suggesting that differential protein expression can further influence disease tropism [80]. Additionally, differences in transcript, protein, and metabolite profiles of parasite isolates with similar genetic makeup can lead to differential virulence and tissue tropism. Such a study on *L*. *(V*.*) braziliensis* isolates from mucosal and cutaneous sites of the same patient revealed the potential role of the prostaglandin synthesis pathway in the differential tropism based on the over-expression of prostaglandin F2 alpha synthase (PGF2s) and heat shock protein 70 (HSP70) in the isolates from the cutaneous site [81]. Such a phenomenon holds potential risk such that a region with atypical CL caused by dermotropic *L*. *donovani* variants could evolve into a focus of systemic manifestation resulting in a VL outbreak [8,80]. Similarly *L*. *tropica* variants with higher virulence, responsible for atypical VL, may revert to a dermotropic form leading to emergence of CL in a region otherwise endemic for VL [4].

Thus, genetic heterogeneity and differential gene expression are critical in determining parasite virulence and the clinical outcome. In this context, it is noteworthy that *L*. *donovani* clinical isolates causing atypical CL cases in Kerala have the same Hsp70 and 6-PGDH gene sequences as the atypical *L*. *donovani* isolates from Sri Lanka, suggesting that the parasite variant in Kerala came from Sri Lanka [9]. Similarly, CL-causing *L*. *donovani* isolates from Himachal Pradesh are reported to be distinct from MON-2 strain based on GPI and gp63 gene sequences [7]. Unraveling genetic variations at the whole genome level and global gene expression studies in a larger number of disease-specific *L*. *donovani* isolates from Kerala and Himachal Pradesh is crucial to our understanding of the genetic basis of atypical CL phenotype in these newly discovered endemic sites in India.

Additionally, patients with an immune-compromised state are known to exhibit atypical disease manifestations with different degrees of parasite metastasis and clinical features, thus implying the significance of host immune status in the disease outcome [74,82]. A review of this variation is beyond the scope of the present article.

## Discussion and conclusion

Leishmaniasis disease complex, especially the systemic form, is a priority for elimination by WHO and local bodies in endemic regions. Despite substantial progress towards VL elimination starting from 2005 in Nepal, Bangladesh, and India, the time frame for its accomplishment in India was extended from 2015 to 2019 with the addition of Bhutan [39–41]. Lack of awareness, poor health service infrastructure, under-reported cases, asymptomatic carriers, PKDL cases as potential parasite reservoirs, and the emergence of drug-resistant parasite variants are major challenges that must be addressed in leishmaniasis control programs. However, one of the most dramatic phenomena that has not been given sufficient consideration is the discovery of new endemic foci with atypical leishmaniasis.

Classical species-specific clinical outcomes are now becoming less valid with the discovery of atypical *Leishmania* variants responsible for atypical clinical outcomes. This review highlights parasite species variants as a key cause of atypical leishmaniasis. Apart from the underlying causative parasite species variants, atypical VL and CL disease often exhibit peculiar variations in terms of disease severity and duration [20,31].

With respect to the scenario in the Indian subcontinent, VL and CL are intruding into newer regions along with findings of atypical leishmaniasis in the known and newer disease territories. While molecularly distinct *L*. *tropica* VL isolates have been demonstrated in northeast India, a rising number of *L*. *donovani*–mediated CL cases are reported in Sri Lanka and in the states of Kerala and Himachal Pradesh in India. In fact, studies on *L*. *donovani* CL isolates from Sri Lanka and Kerala argues for their close genetic lineage. A detailed molecular characterization of clinical isolates from Himachal is still lacking. Based on our work in specific afflicted pockets of Himachal Pradesh, the CL case incidence is on an increase, and the underlying causative parasite are variants of *L*. *donovani* species.

Taken together, it is apparent that parasite variants capable of causing atypical disease manifestations are circulating in different known and newer endemic regions of the globe and the Indian subcontinent. However, it is noteworthy that there is a lack of consensus on the criteria that can be used to characterize and assign parasite isolates as atypical variants in relation to atypical disease manifestation. Limitations on the coverage and depth of studies to distinguish indigenous versus imported disease cases, use of heterogeneous methods to type clinical isolates, the possibility of an inaccurate parasite identification, and technical difficulties in culturing parasite isolates from clinical samples all need to be recognized as challenges. Thus, for a comprehensive epidemiological picture in the understudied endemic regions, multicenter studies incorporating genome-wide typing in relation to biological characterization are required. Studies on vector species and alternate reservoirs are also required for better understanding of region-specific disease transmission and epidemiology.

Irrespective of the above limitations, it is imperative that atypical disease be recognized as a major threat to ongoing leishmaniasis elimination and maintenance programs. Concerted efforts from surveillance bodies, medical staff, and researchers are required to identify and characterize new *Leishmania* isolates from endemic and non-endemic areas to decipher origin and relatedness of region-specific parasite variants. This will help in understanding the biology of the atypical disease phenotype in terms of parasite genetics and will facilitate better monitoring and prediction of transmission patterns in a region specific manner. While the newer geographical niches endemic for the disease warrant wider implementation of the control programs, a continuous monitoring of the disease type and the associated parasite species and their variants should be implemented as part of the ongoing leishmaniasis elimination and maintenance programs.

**Key learning points**

- Atypical leishmaniasis driven by atypical parasite variants is appearing in known and newer endemic regions of the world.
- A particular parasite species can cause one or more disease phenotypes due to genetic heterogeneity of parasite genes.
- Discovery of novel parasite variants should be recognized as an important challenge for leishmaniasis control and elimination.

**Top five papers**

1. Sharma NL, Mahajan VK, Kanga A et al. (2005) Localized cutaneous leishmaniasis due to Leishmania donovani and Leishmania tropica: preliminary findings of the study of 161 new cases from a new endemic focus in Himachal Pradesh, India. The American journal of tropical medicine and hygiene 72: 819–824.
2. Karunaweera N, Pratlong F, Siriwardane H et al (2003) Sri Lankan cutaneous leishmaniasis is caused by Leishmania donovani zymodeme MON-37. Transactions of the Royal Society of Tropical Medicine and Hygiene 97: 380–381.
3. Kumar NP, Srinivasan R, Anish T, Nandakumar G, Jambulingam P (2015) Cutaneous leishmaniasis caused by Leishmania donovani in the tribal population of the Agasthyamala Biosphere Reserve forest, Western Ghats, Kerala, India. Journal of medical microbiology 64: 157–163.
4. Zhang WW, Ramasamy G, McCall L-I et al. (2014) Genetic analysis of Leishmania donovani tropism using a naturally attenuated cutaneous strain. PLoS Pathog 10: e1004244.
5. Krayter L, Bumb RA, Azmi K, et al. (2014) Multilocus microsatellite typing reveals a genetic relationship but, also, genetic differences between Indian strains of Leishmania tropica causing cutaneous leishmaniasis and those causing visceral leishmaniasis. Parasites & vectors 7: 123

## Supporting information

S1 Table Geographical distribution of classical and atypical leishmaniasis with the causative agents in Old and New World countries. (DOCX)

S2 Table Geographical distribution of classical and atypical leishmaniasis with the causative agents in Indian subcontinent. (DOCX)

## References

[1] World Health Organization Global Health Observatory, Leishmaniasis (2017). http://www.who.int/gho/neglected_diseases/leishmaniasis/en/. [cited 2017 Sep 30]

[2] World Health Organization Media Centre, Leishmaniasis (2017). http://www.who.int/mediacentre/factsheets/fs375/en/. [cited 2017 Sep 30]

[3] Alvar J, Vélez ID, Bern C, Herrero M, Desjeux P, et al (2012) Leishmaniasis worldwide and global estimates of its incidence. PloS ONE 7: e35671 10.1371/journal.pone.0035671 22693548 PMC3365071

[4] Khanra S, Datta S, Mondal D, Saha P, Bandopadhyay SK, et al (2012) RFLPs of ITS, ITS1 and hsp70 amplicons and sequencing of ITS1 of recent clinical isolates of Kala-azar from India and Bangladesh confirms the association of L. tropica with the disease. Acta tropica 124: 229–234. 10.1016/j.actatropica.2012.08.017 22960646

[5] Krayter L, Bumb RA, Azmi K, Wuttke J, Malik MD, et al (2014) Multilocus microsatellite typing reveals a genetic relationship but, also, genetic differences between Indian strains of Leishmania tropica causing cutaneous leishmaniasis and those causing visceral leishmaniasis. Parasites & vectors 7: 123. 24666968 10.1186/1756-3305-7-123 PMC3987047

[6] Karunaweera N, Pratlong F, Siriwardane H, Ihalamulla R, Dedet J (2003) Sri Lankan cutaneous leishmaniasis is caused by Leishmania donovani zymodeme MON-37. Transactions of the Royal Society of Tropical Medicine and Hygiene 97: 380–381. 15259461 10.1016/s0035-9203(03)90061-7

[7] Sharma NL, Mahajan VK, Kanga A, Sood A, Katoch VM, et al (2005) Localized cutaneous leishmaniasis due to Leishmania donovani and Leishmania tropica: preliminary findings of the study of 161 new cases from a new endemic focus in Himachal Pradesh, India. The American journal of tropical medicine and hygiene 72: 819–824. 15964970

[8] Zhang WW, Ramasamy G, McCall L-I, Haydock A, Ranasinghe S, et al (2014) Genetic analysis of Leishmania donovani tropism using a naturally attenuated cutaneous strain. PLoS Pathog 10: e1004244 10.1371/journal.ppat.1004244 24992200 PMC4081786

[9] Kumar NP, Srinivasan R, Anish T, Nandakumar G, Jambulingam P (2015) Cutaneous leishmaniasis caused by Leishmania donovani in the tribal population of the Agasthyamala Biosphere Reserve forest, Western Ghats, Kerala, India. Journal of medical microbiology 64: 157–163. 10.1099/jmm.0.076695-0 25480880

[10] McCall L-I, Zhang W-W, Matlashewski G (2013) Determinants for the development of visceral leishmaniasis disease. PLoS Pathog 9: e1003053 10.1371/journal.ppat.1003053 23300451 PMC3536654

[11] Zhang WW, Matlashewski G (2010) Screening Leishmania donovani-specific genes required for visceral infection. Molecular microbiology 77: 505–517. 10.1111/j.1365-2958.2010.07230.x 20545850

[12] Dhiman RC (2014) Emerging vector-borne zoonoses: eco-epidemiology and public health implications in India. Frontiers in public health 2. 10.3389/fpubh.2014.00168 PMC4179687 25325052

[13] Duprey ZH (2006) Canine Visceral Leishmaniasis, United States and Canada, 2000–2003-Volume 12, Number 3—March 2006-Emerging Infectious Disease journal-CDC. 10.3201/eid1203.050811 PMC3291440 16704782

[14] Kumar Bhat N, Ahuja V, Dhar M, Ahmad S, Pandita N, et al (2017) Changing Epidemiology: A New Focus of Kala-azar at High-Altitude Garhwal Region of North India. Journal of tropical pediatrics 63: 104–108. 10.1093/tropej/fmw056 27582128

[15] Raina S, Raina RK, Sharma R, Rana BS, Bodh A, et al (2016) Expansion of visceral leishmaniasis to northwest sub-Himalayan region of India: A case series. Journal of Vector Borne Diseases 53: 188 27353591

[16] Ready P (2010) Leishmaniasis emergence in Europe. Euro surveill 15: 19505 20403308

[17] Siriwardana HD, Karunanayake P, Goonerathne L, Karunaweera N (2017) Emergence of visceral leishmaniasis in Sri Lanka: a newly established health threat. Pathogens and global health: 1–10. 10.1080/20477724.2017.1361564 PMC5694859 28820339

[18] Alborzi A, Pouladfar GR, Fakhar M, Motazedian MH, Hatam GR, et al (2008) Isolation of Leishmania tropica from a patient with visceral leishmaniasis and disseminated cutaneous leishmaniasis, southern Iran. The American journal of tropical medicine and hygiene 79: 435–437. 18784238

[19] Alborzi A, Rasouli M, Shamsizadeh A (2006) Leishmania tropica–isolated patient with visceral leishmaniasis in southern Iran. The American journal of tropical medicine and hygiene 74: 306–307. 16474088

[20] Magill AJ, Grogl M, Gasser RA Jr, Sun W, Oster CN (1993) Visceral infection caused by Leishmania tropica in veterans of Operation Desert Storm. New England Journal of Medicine 328: 1383–1387. 10.1056/NEJM199305133281904 8292114

[21] Mebrahtu Y, Lawyer P, Githure J, Were JB, Muigai R, et al (1989) Visceral leishmaniasis unresponsive to pentostam caused by Leishmania tropica in Kenya. The American journal of tropical medicine and hygiene 41: 289–294. 2552850 10.4269/ajtmh.1989.41.289

[22] Oren R, Schnur L, Yehuda DB, Mayner V, Okon E, et al (1991) Visceral leishmaniasis: a difficult diagnosis and unusual causative agent. Journal of Infectious Diseases 164: 746–749. 1654358 10.1093/infdis/164.4.746

[23] Sarkari B, Ahmadpour NB, Moshfe A, Hajjaran H (2016) Molecular evaluation of a case of visceral leishmaniasis due to Leishmania tropica in Southwestern Iran. Iranian journal of parasitology 11: 126 27095980 PMC4835463

[24] Freundlich E, May M (1972) Visceral leishmaniasis in western Galilee. Epidemiological review of the years 1960–9. Harefuah 83: 223–225. 4650392

[25] Schlein Y, Warburg A, Schnur L, Le Blancq S, Gunders A (1984) Leishmaniasis in Israel: reservoir hosts, sandfly vectors and leishmanial strains in the Negev, Central Arava and along the Dead Sea. Transactions of the Royal Society of Tropical Medicine and Hygiene 78: 480–484. 6385358 10.1016/0035-9203(84)90067-1

[26] Barral A, Pedral-Sampaio D, Grimaldi JG, Momen H, McMahon-Pratt D, et al (1991) Leishmaniasis in Bahia, Brazil: evidence that Leishmania amazonensis produces a wide spectrum of clinical disease. The American journal of tropical medicine and hygiene 44: 536–546. 2063957 10.4269/ajtmh.1991.44.536

[27] de Oliveira JPC, Fernandes F, Cruz AK, Trombela V, Monteiro E, et al (2007) Genetic diversity of Leishmania amazonensis strains isolated in northeastern Brazil as revealed by DNA sequencing, PCR-based analyses and molecular karyotyping. Kinetoplastid biology and disease 6: 5 10.1186/1475-9292-6-5 17584940 PMC1919383

[28] Karamian M, Motazedian MH, Mehrabani D, Gholami K (2007) Leishmania major infection in a patient with visceral leishmaniasis: treatment with Amphotericin B. Parasitology research 101: 1431–1434. 10.1007/s00436-007-0649-x 17659388

[29] Aoun K, Bouratbine A (2014) Cutaneous leishmaniasis in North Africa: a review. Parasite 21. 10.1051/parasite/2014014 PMC3952656 24626301

[30] del Giudice P, Marty P, Lacour JP, Perrin C, Pratlong F, et al (1998) Cutaneous leishmaniasis due to Leishmania infantum: Case reports and literature review. Archives of dermatology 134: 193–198. 9487211 10.1001/archderm.134.2.193

[31] Rioux J, Lanotte G, Maazoun R, Perello R, Pratlong F (1980) Leishmania infantum Nicolle, 1908, the agent of the autochthonous oriental sore. Apropos of the biochemical identification of 2 strains isolated in the eastern Pyrenees. Comptes rendus des seances de l’Academie des sciences Serie D, Sciences naturelles 291: 701–703. 6780226

[32] Knio K, Baydoun E, Tawk R, Nuwayri-Salti N (2000) Isoenzyme characterization of Leishmania isolates from Lebanon and Syria. The American journal of tropical medicine and hygiene 63: 43–47. 11357993 10.4269/ajtmh.2000.63.43

[33] Convit J, Ulrich M, Pérez M, Hung J, Castillo J, et al (2005) Atypical cutaneous leishmaniasis in Central America: possible interaction between infectious and environmental elements. Transactions of the Royal Society of Tropical Medicine and Hygiene 99: 13–17. 10.1016/j.trstmh.2004.02.005 15550256

[34] Elamin E, Guizani I, Guerbouj S, Gramiccia M, El Hassan A, et al (2008) Identification of Leishmania donovani as a cause of cutaneous leishmaniasis in Sudan. Transactions of the Royal Society of Tropical Medicine and Hygiene 102: 54–57. 10.1016/j.trstmh.2007.10.005 18037149

[35] Mebrahtu YB, Van Eys G, Guizani I, Lawyer PG, Pamba H, et al (1993) Human cutaneous leishmaniasis caused by Leishmania donovani sl in Kenya. Transactions of the Royal Society of Tropical Medicine and Hygiene 87: 598–601. 8266420 10.1016/0035-9203(93)90101-u

[36] Khatri ML, Di Muccio T, Fiorentino E, Gramiccia M (2016) Ongoing outbreak of cutaneous leishmaniasis in northwestern Yemen: clinicoepidemiologic, geographic, and taxonomic study. International Journal of Dermatology 55: 1210–1218. 10.1111/ijd.13310 27419356

[37] Nakkash-Chmaisse H, Makki R, Nahhas G, Knio K, Nuwayri-Salti N (2011) Detection of Leishmania parasites in the blood of patients with isolated cutaneous leishmaniasis. International Journal of Infectious Diseases 15: e491–e494. 10.1016/j.ijid.2011.03.022 21621442

[38] Naucke T, Schmitt C (2004) Is leishmaniasis becoming endemic in Germany? International Journal of Medical Microbiology Supplements 293: 179–181. 10.1016/s1433-1128(04)80036-6 15147005

[39] WHO (2005–2015) Regional strategic framework for elimination of kala-azar from the South-East Asia region (2005–2015). New Delhi: WHO Regional Office for South-East Asia.

[40] Olliaro PL, Shamsuzzaman TA, Marasini B, Dhariwal A, Be-Nazir A, et al (2017) Investments in Research and Surveillance Are Needed to Go Beyond Elimination and Stop Transmission of Leishmania in the Indian Subcontinent. PLoS Negl Trop Dis 11: e0005190 10.1371/journal.pntd.0005190 28125596 PMC5268387

[41] Hirve S, Boelaert M, Matlashewski G, Mondal D, Arana B, et al (2016) Transmission dynamics of visceral leishmaniasis in the indian subcontinent–a systematic literature review. PLoS Negl Trop Dis 10: e0004896 10.1371/journal.pntd.0004896 27490264 PMC4973965

[42] World Health Organization, 2010|2014, Leishmaniasis: Country Profiles (2016 July). http://www.who.int/leishmaniasis/burden/Country_profiles/en/. [cited 2017 Sep 30]

[43] Schwarz D, Andrews J, Gauchan B (2011) Visceral leishmaniasis in far western Nepal: another case and concerns about a new area of endemicity. The American journal of tropical medicine and hygiene 84: 508–508. 10.4269/ajtmh.2011.11-0021 21363996 PMC3042834

[44] Ostyn B, Uranw S, Bhattarai NR, Das ML, Rai K, et al (2015) Transmission of Leishmania donovani in the hills of Eastern Nepal, an outbreak investigation in Okhaldhunga and Bhojpur districts. PLoS Negl Trop Dis 9: e0003966 10.1371/journal.pntd.0003966 26252494 PMC4529159

[45] KalaCORE Where we work, Bangladesh (2017 July). http://www.kalacore.org/where-we-work/bangladesh. [cited 2017 Sep 30]

[46] Mondal D, Nasrin KN, Huda MM, Kabir M, Hossain MS, et al (2010) Enhanced case detection and improved diagnosis of PKDL in a Kala-azar-endemic area of Bangladesh. PLoS Negl Trop Dis 4: e832 10.1371/journal.pntd.0000832 20957193 PMC2950134

[47] Yangzom T, Cruz I, Bern C, Argaw D, den Boer M, et al (2012) Endemic transmission of visceral leishmaniasis in Bhutan. The American journal of tropical medicine and hygiene 87: 1028–1037. 10.4269/ajtmh.2012.12-0211 23091191 PMC3516070

[48] Ranasinghe S, Zhang W-W, Wickremasinghe R, Abeygunasekera P, Chandrasekharan V, et al (2012) Leishmania donovani zymodeme MON-37 isolated from an autochthonous visceral leishmaniasis patient in Sri Lanka. Pathogens and global health 106: 421–424. 10.1179/2047773212Y.0000000054 23265615 PMC4001626

[49] Sharma U, Redhu NS, Mathur P, Singh S (2007) Re-emergence of visceral leishmaniasis in Gujarat, India. Journal of vector borne diseases 44: 230 17896628

[50] Dhiman RC, Pahwa S, Dhillon G, Dash AP (2010) Climate change and threat of vector-borne diseases in India: are we prepared? Parasitology Research 106: 763–773. 10.1007/s00436-010-1767-4 20155369

[51] Dey A, Sharma U, Singh S (2007) First case of indigenous visceral leishmaniasis from central India. The American journal of tropical medicine and hygiene 77: 95–98. 17620636

[52] Nandedkar SS, Malukani K, Varma A (2011) Maiden visit of visceral leishmaniasis to Malwa region. The Journal of communicable diseases 43: 233–235. 23781638

[53] Kaushal K, Veena M (2008) Two cases of Kala-azar in Haryana with no evidence of local transmission. 19127677

[54] Kesavan A, Parvathy V, Thomas S, Sudha S (2003) Indigenous visceral leishmaniasis: two cases from Kerala. Indian pediatrics 40: 373–373. 12736419

[55] Ahmad S, Chandra H, Bhat NK, Dhar M, Shirazi N, et al (2016) North Indian state of Uttarakhand: a new hothouse of visceral leishmaniasis. Tropical doctor 46: 111–113. 10.1177/0049475515609245 26466848

[56] Mahajan SK, Machhan P, Kanga A, Thakur S, Sharma A, et al (2004) Kala-azar at high altitude. Journal of Communicable Diseases 36: 117 16295673

[57] Raina S, Mahesh D, Kaul R, Satinder KS, Gupta D, et al (2009) A new focus of visceral leishmaniasis in the Himalayas, India. Journal of vector borne diseases 46: 303 19959858

[58] Chandra H, Chandra S, Bhat NK, Sharma A (2011) Clinicohaematological profile of infections in bone marrow–single centre experience in North Himalayan region of India. Hematology 16: 255–257. 10.1179/102453311X13025568941844 21756544

[59] Chufal SS, Pant P, Chachra U, Singh P, Thapliyal N, et al. (2016) Role of Haematological Changes in Predicting Occurrence of Leishmaniasis-A Study in Kumaon Region of Uttarakhand. 10.7860/JCDR/2016/15438.7885 PMC4948406 27437230

[60] Bhat NK, Ahuja V, Dhar M, Ahmad S, Pandita N, et al (2016) Changing Epidemiology: A New Focus of Kala-azar at High-Altitude Garhwal Region of North India. Journal of Tropical Pediatrics: fmw056. 10.1093/tropej/fmw056 27582128

[61] Sharma NL, Mahajan VK, Ranjan N, Verma GK, Negi AK, et al (2009) The sandflies of the Satluj river valley, Himachal Pradesh (India): some possible vectors of the parasite causing human cutaneous and visceral leishmaniases in this endemic focus. Journal of vector borne diseases 46: 136 19502693

[62] Mahajan D, Bhat M, Singh J, Hans D (2009) Visceral Leishmaniasis In A Native Kashmiri Boy.

[63] Bhat K, Pandita K, Khajuria A, Wani S (2014) Visceral leishmaniasis (kalazar) migrating West: A new autochthonous case from sub-Himalayas. Indian journal of medical microbiology 32: 94 10.4103/0255-0857.124344 24399404

[64] Kumar R, Bumb RA, Ansari NA, Mehta RD, Salotra P (2007) Cutaneous leishmaniasis caused by Leishmania tropica in Bikaner, India: parasite identification and characterization using molecular and immunologic tools. The American journal of tropical medicine and hygiene 76: 896–901. 17488912

[65] Kaul N, Gupta V, Bhardwaj S, Dogra D, Dogra N (2016) A new focus of cutaneous leishmaniasis in Jammu division of Jammu and Kashmir State, India. Indian Journal of Dermatology, Venereology, and Leprology 82: 145. 10.4103/0378-6323.175930 26924403

[66] Wani GM, Ahmad SM, Khursheed B (2015) Clinical study of cutaneous leishmaniasis in the Kashmir Valley. Indian dermatology online journal 6: 387–392. 10.4103/2229-5178.169732 26753136 PMC4693348

[67] Kumar R, Ansari NA, Avninder S, Ramesh V, Salotra P (2008) Cutaneous leishmaniasis in Nepal: Leishmania major as a cause. Transactions of the Royal Society of Tropical Medicine and Hygiene 102: 202–203. 10.1016/j.trstmh.2007.10.017 18177679

[68] Basher A, Nath P, Dey T, Sayeed AA, Faiz MA, et al (2017) Cutaneous leishmaniasis in immigrant workers returning to Bangladesh–an emerging problem. Travel Medicine and Infectious Disease. 10.1016/j.tmaid.2017.05.013 28591658

[69] Karunaweera ND (2009) Leishmania donovani causing cutaneous leishmaniasis in Sri Lanka: a wolf in sheep’s clothing? Trends in parasitology 25: 458–463. 10.1016/j.pt.2009.07.002 19734098

[70] Nandha B, Srinivasan R, Jambulingam P (2014) Cutaneous leishmaniasis: knowledge, attitude and practices of the inhabitants of the Kani forest tribal settlements of Tiruvananthapuram district, Kerala, India. Health education research: cyu064. 10.1093/her/cyu064 25325998

[71] Srinivasan R, Kumar NP, Jambulingam P (2016) Detection of natural infection of Leishmania donovani (Kinetoplastida: Trypanosomatidae) in Phlebotomus argentipes (Diptera: Psychodidae) from a forest ecosystem in the Western Ghats, India, endemic for cutaneous leishmaniasis. Acta tropica 156: 95–99. 10.1016/j.actatropica.2016.01.010 26774685

[72] Sharma NL, Mahajan VK, Negi AK, Verma GK (2009) The rK39 immunochromatic dipstick testing: a study for K39 seroprevalence in dogs and human leishmaniasis patients for possible animal reservoir of cutaneous and visceral leishmaniasis in endemic focus of Satluj river valley of Himachal Pradesh (India). Indian Journal of Dermatology, Venereology, and Leprology 75: 52. 10.4103/0378-6323.45221 19172032

[73] Sharma R, Mahajan V, Sharma N, Sharma A (2003) A new focus of cutaneous leishmaniasis in Himachal Pradesh (India). Indian Journal of Dermatology, Venereology, and Leprology 69: 170. 17642870

[74] Bains A, Vedant D, Gupta P, Tegta G (2016) Unusual presentation of mucocutaneous leishmaniasis in HIV-infected patient. Indian Journal of Sexually Transmitted Diseases 37: 193. 10.4103/2589-0557.192118 PMC5111308 27890957

[75] Srivastava P, Singh T, Sundar S (2011) Genetic heterogeneity in clinical isolates of Leishmania donovani from India. Journal of clinical microbiology 49: 3687–3690. 10.1128/JCM.00729-11 21865422 PMC3187323

[76] Downing T, Imamura H, Decuypere S, Clark TG, Coombs GH, et al (2011) Whole genome sequencing of multiple Leishmania donovani clinical isolates provides insights into population structure and mechanisms of drug resistance. Genome research 21: 2143–2156. 10.1101/gr.123430.111 22038251 PMC3227103

[77] Zackay A, Cotton JA, Sanders M, Hailu A, Nasereddin A, et al (2018) Genome wide comparison of Ethiopian Leishmania donovani strains reveals differences potentially related to parasite survival. PLoS Genet 14: e1007133 10.1371/journal.pgen.1007133 29315303 PMC5777657

[78] Imamura H, Downing T, Van den Broeck F, Sanders MJ, Rijal S, et al (2016) Evolutionary genomics of epidemic visceral leishmaniasis in the Indian subcontinent. Elife 5: e12613 10.7554/eLife.12613 27003289 PMC4811772

[79] Gupta AK, Srivastava S, Singh A, Singh S (2015) De novo whole-genome sequence and annotation of a Leishmania strain isolated from a case of post-kala-azar dermal leishmaniasis. Genome announcements 3: e00809–00815. 10.1128/genomeA.00809-15 26184949 PMC4505137

[80] McCall L-I, Zhang W-W, Dejgaard K, Atayde VD, Mazur A, et al (2015) Adaptation of Leishmania donovani to cutaneous and visceral environments: in vivo selection and proteomic analysis. Journal of proteome research 14: 1033–1059. 10.1021/pr5010604 25536015

[81] Alves-Ferreira EV, Toledo JS, De Oliveira AH, Ferreira TR, Ruy PC, et al (2015) Differential gene expression and infection profiles of cutaneous and mucosal Leishmania braziliensis isolates from the same patient. PLoS Negl Trop Dis 9: e0004018 10.1371/journal.pntd.0004018 26366580 PMC4569073

[82] Santos-Oliveira JR, Da-Cruz AM, Pires LH, Cupolillo E, Kuhls K, et al (2011) Atypical Lesions as a Sign of Cutaneous Dissemination of Visceral Leishmaniasis in a Human Immunodeficiency Virus–Positive Patient Simultaneously Infected by Two Viscerotropic Leishmania Species. The American journal of tropical medicine and hygiene 85: 55–59. 10.4269/ajtmh.2011.10-0398 21734124 PMC3122343

