# Supplementary material for: Atypical leishmaniasis: A global perspective with emphasis on the Indian subcontinent
Source: PLoS Negl Trop Dis. 2018 Sep 27;12(9):e0006659. doi: 10.1371/journal.pntd.0006659 (PMC6159859; doi:10.1371/journal.pntd.0006659)
Supplement: S1 Table — (DOCX) [file pntd.0006659.s001.docx]

**S1 Table: Geographical distribution of Classical and Atypical Leishmaniasis with the causative agents in Old and New World countries**

|  | **Classical Leishmaniasis** | | | | | | **Atypical Leishmaniasis** | | |
| --- | --- | --- | --- | --- | --- | --- | --- | --- | --- |
| **Country** | **VL Disease status** | **Species involved** | **CL disease status** | **Species involved** | **Disease Endemicity (VL, CL or Both)** | **References** | **Disease form** | **Species involved** | **References** |
| **Old World Countries** | | | | | | | | | |
| **Afghanistan** | Endemic | *L. infantum* | Endemic | *L. tropica,*  *L. major* | VL and CL | [[1-3](#_ENREF_1)] |  |  |  |
| **Albania** | Endemic | *L. infantum* | Endemic | *L. major* | VL and CL | [[1-3](#_ENREF_1)] | CL | *L. infantum* | [[3](#_ENREF_3)] |
| **Algeria** | Endemic | *L. infantum* | Endemic | *L. killicki,*  *L. major* | VL and CL | [[1-3](#_ENREF_1)] | CL | *L. infantum* | [[3](#_ENREF_3),[4](#_ENREF_4)] |
| **Armenia** | Endemic | *L. infantum* | Endemic |  | VL and CL | [[1-3](#_ENREF_1)] | CL | *L. infantum* | [[3](#_ENREF_3)] |
| **Azerbaijan** | Endemic | *L. infantum* | Endemic | *L. major,*  *L. killicki* | VL and CL | [[1-3](#_ENREF_1)] | CL | *L. infantum* | [[3](#_ENREF_3)] |
| **Bangladesh** | Endemic | *L. donovani* |  |  | VL | [[1-3](#_ENREF_1)] |  |  |  |
| **Bhutan** | Endemic and recent geographic extension | *L. donovani* |  |  | VL | [[1-3](#_ENREF_1)] |  |  |  |
| **Bosnia and Herzegovina** | Endemic | *L infantum* | Endemic |  | VL and CL | [[1-3](#_ENREF_1)] | CL | *L. infantum* | [[3](#_ENREF_3)] |
| **Bulgaria** | Endemic | *L. infantum* | Endemic |  | VL and CL | [[1-3](#_ENREF_1)] | CL | *L. infantum* | [[3](#_ENREF_3)] |
| **Burkina Faso** |  |  | Endemic | *L. major* | CL | [[1](#_ENREF_1),[2](#_ENREF_2)] |  |  |  |
| **Cameroon** | Previously reported cases | Unknown | Endemic | *L. major* | VL and CL | [[1-3](#_ENREF_1)] |  |  |  |
| **Central African Republic** | Previously reported cases | *L. infantum* | Previously reported cases |  | VL and CL | [[1](#_ENREF_1),[2](#_ENREF_2)] |  |  |  |
| **Chad** | Endemic | Unknown | Endemic | *L. major* | VL and CL | [[1-3](#_ENREF_1)] |  |  |  |
| **China** | Endemic | *L. infantum, L. donovani* | Endemic |  | VL and CL | [[1-3](#_ENREF_1)] | CL | *L. infantum* | [[3](#_ENREF_3)] |
| **Croatia** | Endemic | *L. infantum* | Endemic |  | VL and CL | [[1-3](#_ENREF_1)] | CL | *L. infantum* | [[3](#_ENREF_3)] |
| **Cyprus** | Endemic | *L. infantum, L. donovani* | Endemic |  | VL and CL | [[1-3](#_ENREF_1)] | CL | *L. infantum,*  *L. donovani* | [[3](#_ENREF_3),[5](#_ENREF_5)] |
| **Cote d’Lvoire** | Endemic | Unknown | Endemic | *unknown* | VL and CL | [[1-3](#_ENREF_1)] |  |  |  |
| **DR Congo** | Endemic | Unknown | Endemic | *unknown* | VL and CL | [[1-3](#_ENREF_1)] |  |  |  |
| **Djibouti** | Endemic | *L. donovani* | Endemic | *unknown* | VL and CL | [[1-3](#_ENREF_1)] |  |  |  |
| **Egypt** | Endemic | *L. infantum* | Endemic | *L. major,*  *L. tropica* | VL and CL | [[1-3](#_ENREF_1)] |  |  |  |
| **Eritrea** | Endemic | Unknown | Endemic | *unknown* | VL and CL | 1, 32 |  |  |  |
| **Ethiopia** | Endemic | *L.donovani* | Endemic | *L. aethiopica,*  *L. major,*  *L. tropica* | VL and CL | [[1-3](#_ENREF_1)] | CL | *L. donovani* | [[6](#_ENREF_6)] |
| **France** | Endemic | *L.infantum* | Endemic |  | VL and CL | [[1-3](#_ENREF_1)] | CL | *L.infantum* | [[3](#_ENREF_3),[5](#_ENREF_5)] |
| **Gambia** | Previously reported cases | *L. infantum* | Previously reported cases |  | VL and CL | [[1](#_ENREF_1),[2](#_ENREF_2)] |  |  |  |
| **Georgia** | Endemic | *L. infantum* | Endemic | *L. major* | VL and CL | [[1-3](#_ENREF_1)] |  |  |  |
| **Ghana** |  |  | Endemic | *L. major,* | CL | [[1-3](#_ENREF_1)] |  |  |  |
| **Greece** | Endemic | *L. infantum* | Endemic | *L. tropica* | VL and CL | [[1-3](#_ENREF_1)] | CL | *L. infantum* | [[3](#_ENREF_3),[7](#_ENREF_7)] |
| **Guinea** |  |  | Endemic | *L. major* | CL | [[1](#_ENREF_1),[2](#_ENREF_2)] |  |  |  |
| **Guinea Bissau** |  |  | Endemic | *L. major* | CL | [[1](#_ENREF_1),[2](#_ENREF_2)] |  |  |  |
| **India** | Endemic | *L. donovani* | Endemic | *L. major,*  *L. tropica* | VL and CL | [[1-3](#_ENREF_1)] | VL and CL | *L. tropica,*  *L. donovani* | [[8-10](#_ENREF_8)] |
| **Iran** | Endemic | *L. infantum* | Endemic | *L. major,*  *L. tropica* | VL and CL | [[1-3](#_ENREF_1)] | VL | *L. major,*  *L. tropica* | [[11-13](#_ENREF_11)] |
| **Iraq** | Endemic | *L. donovani, L. infantum* | Endemic | *L. major,*  *L. tropica* | VL and CL | [[1-3](#_ENREF_1)] |  |  |  |
| **Israel** | Endemic | *L. infantum* | Endemic | *L. major,*  *L. tropica* | VL and CL | [[1-3](#_ENREF_1)] | VL and CL | *L. donovani,*  *L. infantum,*  *L. tropica* | [[3](#_ENREF_3),[14](#_ENREF_14)] |
| **Italy** | Endemic | *L. infantum* | Endemic |  | VL and CL | [[1-3](#_ENREF_1)] | CL | *L infantum* | [[3](#_ENREF_3),[5](#_ENREF_5),[15](#_ENREF_15)] |
| **Jordan** | Endemic | *L. infantum* | Endemic | *L. major,*  *L. tropica* | VL and CL | [[1-3](#_ENREF_1)] |  |  |  |
| **Kazakhstan** | Endemic | *L. infantum* | Endemic | *L. major* | VL and CL | [[1-3](#_ENREF_1)] |  |  |  |
| **Kenya** | Endemic | *L. donovani* | Endemic | *L. tropica,*  *L. aethiopica,*  *L. major* | VL and CL | [[1-3](#_ENREF_1)] | VL and CL | *L. tropica,*  *L. donovani* | [[16](#_ENREF_16),[17](#_ENREF_17)] |
| **Kuwait** |  |  | Endemic | *L. major* | CL | [[1](#_ENREF_1),[2](#_ENREF_2)] |  |  |  |
| **Kyrgystan** | Endemic | *L. infantum* | Endemic |  | VL and CL | [[1](#_ENREF_1),[2](#_ENREF_2)] | CL | *L. infantum* | [[3](#_ENREF_3)] |
| **Lebanon** | Endemic | *L. infantum* | Endemic |  | VL and CL | [[1-3](#_ENREF_1)] | CL | *L. infantum* | [[3](#_ENREF_3),[18](#_ENREF_18)] |
| **Libya** | Endemic | *L. infantum* | Endemic | *L. major,*  *L. killicki* | VL and CL | [[1-3](#_ENREF_1)] | CL | *L. infantum* | [[3](#_ENREF_3),[19](#_ENREF_19)] |
| **Malawi** |  |  | Endemic | *Unknown* | CL | [[1](#_ENREF_1)] |  |  |  |
| **Mali** |  |  | Endemic | *L. major* | CL | [[1-3](#_ENREF_1)] |  |  |  |
| **Malta** | Endemic | *L. infantum* | Endemic |  | VL and CL | [[1-3](#_ENREF_1)] | CL | *L. infantum* | [[3](#_ENREF_3),[5](#_ENREF_5)] |
| **Mauritania** | Endemic | *L. infantum* | Endemic | *L. major* | VL and CL | [[1-3](#_ENREF_1)] | CL | *L. infantum* | [[3](#_ENREF_3)] |
| **Monaco** | Endemic | *L. infantum* | Endemic |  | VL and CL | [[1](#_ENREF_1),[2](#_ENREF_2)] |  |  |  |
| **Mongolia** |  |  | Previously reported cases | *L. major* | CL | [[1](#_ENREF_1),[2](#_ENREF_2)] |  |  |  |
| **Montenegro** | Endemic | *L. infantum* | Endemic |  | VL and CL | [[1-3](#_ENREF_1)] |  |  |  |
| **Morocco** | Endemic | *L. infantum* | Endemic | *L. major,*  *L. tropica* | VL and CL | [[1-3](#_ENREF_1)] | CL | *L infantum* | [[3](#_ENREF_3),[19](#_ENREF_19)] |
| **Namibia** |  |  | Endemic | *L. tropica* | CL | [[1](#_ENREF_1),[2](#_ENREF_2)] |  |  |  |
| **Nepal** | Endemic | *L. donovani* | Previously reported cases |  | VL, CL | [[1-3](#_ENREF_1),[20](#_ENREF_20)] |  |  |  |
| **Niger** | Endemic | Unknown | Endemic | *L. major* | VL and CL | [[1](#_ENREF_1),[2](#_ENREF_2)] |  |  |  |
| **Nigeria** | Previously reported cases | Unknown | Endemic | *L. major* | VL and CL | [[1-3](#_ENREF_1)] |  |  |  |
| **Oman** | Endemic | *L. infantum* | Endemic | *L. major* | VL and CL | [[1-3](#_ENREF_1)] |  |  |  |
| **Pakistan** | Endemic | *L. infantum* | Endemic | *L. major,*  *L. tropica* | VL and CL | [[1-3](#_ENREF_1)] |  |  |  |
| **Palestine** |  | *L.infantum* |  | *L. major,*  *L.tropica* | VL and CL | [[2](#_ENREF_2),[3](#_ENREF_3),[21](#_ENREF_21)] |  |  |  |
| **Portugal** | Endemic | *L. infantum* | Endemic |  | VL and CL | [[1-3](#_ENREF_1)] | CL | *L. infantum* | [[3](#_ENREF_3),[22](#_ENREF_22)] |
| **Romania** | Endemic | *L. infantum* |  |  | VL | [[1](#_ENREF_1),[2](#_ENREF_2)] |  |  |  |
| **Saudi Arabia** | Endemic | *L.infantum, L.donovani* | Endemic | *L. major,*  *L.tropica* | VL and CL | [[1-3](#_ENREF_1)] | VL | *L. tropica* | [[14](#_ENREF_14)] |
| **Senegal** | Endemic | *L.infantum* | Endemic | *L. major* | VL and CL | [[1-3](#_ENREF_1)] |  |  |  |
| **Slovenia** | Endemic | *L. infantum* | Endemic |  | VL and CL | [[1](#_ENREF_1),[3](#_ENREF_3)] | CL | *L. infantum* | [[3](#_ENREF_3)] |
| **Somalia** | Endemic | *L.donovani* |  |  | VL | [[1-3](#_ENREF_1)] |  |  |  |
| **Spain** | Endemic | *L.infantum* | Endemic |  | VL and CL | [[1-3](#_ENREF_1)] | CL | *L.infantum* | [[3](#_ENREF_3)] |
| **Sri Lanka** | Previously reported cases and Newer disease sites discovered | *L.donovani* | Endemic and Newer disease sites discovered | *L.donovani* | VL and CL | [[1-3](#_ENREF_1)] | CL | *L.donovani* | [[3](#_ENREF_3),[23](#_ENREF_23),[24](#_ENREF_24)] |
| **South Africa** |  |  | Previously reported cases | *Unknown* | CL | [[3](#_ENREF_3),[25](#_ENREF_25)] |  |  |  |
| **South Sudan** | Endemic | *L.donovani* |  |  | VL | [[1](#_ENREF_1),[3](#_ENREF_3)] |  |  |  |
| **Sudan** | Endemic | *L.infantum, L.donovani* | Endemic | *L. major* | VL and CL | [[1-3](#_ENREF_1)] | CL | *L.donovani* | [[26](#_ENREF_26)] |
| **Syrian Arab Republic** | Endemic | *L. infantum* | Endemic | *L. major,*  *L.tropica* | VL and CL | [[1-3](#_ENREF_1)] | CL | *L. infantum* | [[18](#_ENREF_18)] |
| **Taiwan, China** |  |  |  | *L. tropica* | CL | [[3](#_ENREF_3),[27](#_ENREF_27),[28](#_ENREF_28)] |  |  |  |
| **Tajikistan** | Endemic | *Unknown* | Endemic | *Unknown* | VL and CL | [[1](#_ENREF_1),[3](#_ENREF_3)] |  |  |  |
| **Thailand** | Endemic | *L. siamensis* | Endemic | *L. siamensis* | VL and CL | [[1](#_ENREF_1),[3](#_ENREF_3),[29](#_ENREF_29)] |  |  |  |
| **The Former Yugoslav Republic of Macedonia** | Endemic | *L.infantum* | Endemic |  | VL and CL | [[1-3](#_ENREF_1)] | CL | *L.infantum* | [[3](#_ENREF_3)] |
| **Tunisia** | Endemic | *L.infantum* | Endemic | *L. major,*  *L. killicki* | VL and CL | [[1-3](#_ENREF_1)] | CL | *L.infantum* | [[3](#_ENREF_3),[30](#_ENREF_30)] |
| **Turkey** | Endemic | *L.infantum* | Endemic | *L. tropica* | VL and CL | [[1-3](#_ENREF_1)] | CL | *L.infantum* | [[3](#_ENREF_3)] |
| **Turkmenistan** | Endemic | *L. infantum* | Endemic | *L. major,*  *L. tropica* | VL and CL | [[1-3](#_ENREF_1)] |  |  |  |
| **Uganda** | Endemic | *L. donovani* |  |  | VL | [[1-3](#_ENREF_1)] | CL | *L. donovani* | [[3](#_ENREF_3)] |
| **Ukraine** | Endemic | *L.infantum, L. donovani* | Previously reported cases |  | VL and CL | [[1-3](#_ENREF_1)] |  |  |  |
| **Uzbekistan** | Endemic | *L. infantum* | Endemic | *L. major,*  *L. tropica* | VL and CL | [[1-3](#_ENREF_1)] |  |  |  |
| **Yemen** | Endemic | *L.infantum,*  *L. donovani* | Endemic | *L. major,*  *L. tropica* | VL and CL | [[1-3](#_ENREF_1)] | CL | *L. donovani* | [[31](#_ENREF_31),[32](#_ENREF_32)] |
| **Country** | **VL Disease status** | **Species involved** | **CL disease status** | **Species involved** | **Disease Endemicity (VL, CL or Both)** | **References** | **Disease form** | **Species involved** | **References** |
| **New World Countries** | | | | | | | | | |
| **Argentina** | Endemic | *L. infantum* | Endemic | *L. guyanensis,*  *L. amazonensis,*  *L. braziliensis* | VL and CL | [[1-3](#_ENREF_1)] |  |  |  |
| **Belize** |  |  | Endemic | *L. braziliensis,*  *L. mexicana* | CL | [[1](#_ENREF_1),[2](#_ENREF_2)] |  |  |  |
| **Bolvia** | Endemic | *L. infantum* | Endemic | *L. braziliensis,*  *L.amazonensis,*  *L. guyanensis,*  *L. lainsoni* | VL and CL | [[1-3](#_ENREF_1)] |  |  |  |
| **Brazil** | Endemic | *L. infantum* | Endemic | *L. guyanensis,*  *L. amazonensis,*  *L. braziliensis* | VL and CL | [[1-3](#_ENREF_1)] | VL and CL | *L.infantum /*  *L. chagasi*  *L.amazonensis* | [[33](#_ENREF_33),[34](#_ENREF_34)] |
| **Colombia** | Endemic | *L. infantum* | Endemic | *L. braziliensis,*  *L. panamensis,*  *L. colombiensis,*  *L. amazonensis,*  *L. mexicana* | VL and CL | [[1-3](#_ENREF_1)] | CL | *L. infantum/*  *L. chagasi* | [[35](#_ENREF_35)] |
| **Costa Rica** |  |  | Endemic | *L. panamensis,*  *L. Mexicana,*  *L. braziliensis* | CL | [[1-3](#_ENREF_1)] | CL | *L. infantum/*  *L. chagasi* | [[3](#_ENREF_3),[36](#_ENREF_36)] |
| **Dominican republic** |  |  | Endemic | Unknown | CL | [[1](#_ENREF_1),[3](#_ENREF_3)] |  |  |  |
| **Ecuador** |  |  | Endemic | *L. braziliensis,*  *L. panamensis,*  *L. guyanensis,*  *L. amazonensis,*  *L. mexicana* | CL | [[1-3](#_ENREF_1)] |  |  |  |
| **El Salvador** | Endemic | *L. infantum* | Endemic |  | VL and CL | [[1-3](#_ENREF_1)] | CL | *L.infantum/*  *L. chagasi* | [[3](#_ENREF_3),[5](#_ENREF_5),[37](#_ENREF_37)] |
| **French Guiana** |  |  |  | *L. guyanensis,*  *L. braziliensis,*  *L. amazonensis,*  *L. naiffi* | CL | [[2](#_ENREF_2),[3](#_ENREF_3),[38](#_ENREF_38)] |  |  |  |
| **Guatemala** | Endemic | *L. infantum* | Endemic | *L. braziliensis,*  *L. panamensis,*  *L. mexicana* | VL and CL | [[1-3](#_ENREF_1)] |  |  |  |
| **Guyana** |  |  | Endemic | *L.guyanensis* | CL | [[1-3](#_ENREF_1)] |  |  |  |
| **Honduras** | Endemic | *L.infantum* | Endemic | *L.panamensis,*  *L. braziliensis* | VL and CL | [[1-3](#_ENREF_1)] | CL | *L. infantum/*  *L. chagasi* | [[3](#_ENREF_3),[5](#_ENREF_5),[37](#_ENREF_37)] |
| **Mexico** | Endemic | *L. infantum* | Endemic | *L. braziliensis,*  *L. mexicana* | VL and CL | [[1-3](#_ENREF_1)] |  |  |  |
| **Nicaragua** | Endemic | *L. infantum* | Endemic | *L. panamensis,*  *L. braziliensis* | VL and CL | [[1-3](#_ENREF_1)] | CL | *L. infantum/*  *L. chagasi* | [[3](#_ENREF_3),[39](#_ENREF_39)] |
| **Panama** |  |  | Endemic | *L. panamensis,*  *L. braziliensis,*  *L. colombiensis* | CL | [[1-3](#_ENREF_1)] |  |  |  |
| **Paraguay** | Endemic | *L. infantum* | Endemic | *L. braziliensis* | VL and CL | [[1-3](#_ENREF_1)] |  |  |  |
| **Peru** |  |  | Endemic | *L. peruviana,*  *L. amazonensis,*  *L. guyanensis,*  *L. braziliensis* | CL | [[1-3](#_ENREF_1)] |  |  |  |
| **Suriname** |  |  | Endemic | *L.guyanensis,*  *L. amazonensis,*  *L.lainsoni* | CL | [[1-3](#_ENREF_1)] |  |  |  |
| **USA** |  |  | Newer disease sites discovered | *L. mexicana* | CL | [[1](#_ENREF_1),[3](#_ENREF_3),[40](#_ENREF_40)] |  |  |  |
| **Venezuela** | Endemic | *L. infantum* | Endemic | *L. braziliensis,*  *L. colombiensis,*  *L. venezuelensis,*  *L. amazonensis,*  *L. guyanensis* | VL and CL | [[1-3](#_ENREF_1)] | CL | *L. infantum/*  *L. chagasi* | [[41](#_ENREF_41)] |
| **Zambia** | Endemic | *Unknown* |  |  | VL | [[1](#_ENREF_1),[3](#_ENREF_3)] |  |  |  |

**Major information in the table adapted from Ref 1, Ref 2 and Ref 3**

**Endemic:** A country is classified as endemic if at least one autochthonous case has been reported and the whole cycle of transmission has been demonstrated somewhere in that country (Ref 1).

**Previously reported cases:** A country is classified as having previously reported cases if at least one autochthonous case has been reported but the whole cycle of transmission has not been demonstrated in that country (Ref 1).

*L. infantum* and *L. chagasi* considered synonymous

**References:**

1. World Health Organization (2017) Global Health Observatory, Leishmaniasis (2017 Sep). Available from <http://www.who.int/gho/neglected_diseases/leishmaniasis/en/>.

2. Cantacessi C, Dantas-Torres F, Nolan MJ, Otranto D (2015) The past, present, and future of Leishmania genomics and transcriptomics. Trends in parasitology 31: 100-108.

3. Alvar J, Vélez ID, Bern C, Herrero M, Desjeux P, et al. (2012) Leishmaniasis worldwide and global estimates of its incidence. PloS one 7: e35671.

4. Harrat Z, Pratlong F, Belazzoug S, Dereure J, Deniau M, et al. (1996) Leishmania infantum and L. major in Algeria. Transactions of the Royal Society of Tropical Medicine and Hygiene 90: 625-629.

5. del Giudice P, Marty P, Lacour JP, Perrin C, Pratlong F, et al. (1998) Cutaneous leishmaniasis due to Leishmania infantum: Case reports and literature review. Archives of dermatology 134: 193-198.

6. Gelanew T, Hurissa Z, Diro E, Kassahun A, Kuhls K, et al. (2011) Disseminated Cutaneous Leishmaniasis Resembling Post-Kala-Azar Dermal Leishmaniasis Caused by Leishmania donovani in Three Patients Co-Infected with Visceral Leishmaniasis and Human Immunodeficiency Virus/Acquired Immunodeficiency Syndrome in Ethiopia. The American Journal of Tropical Medicine and Hygiene 84: 906-912.

7. Frank C, Hadziandoniou M, Pratlong F, Garifallou A, Rioux JA (1993) Leishmania tropica and Leishmania infantum responsible for cutaneous leishmaniasis in Greece: sixteen autochthonous cases. Trans R Soc Trop Med Hyg 87.

8. Krayter L, Bumb RA, Azmi K, Wuttke J, Malik MD, et al. (2014) Multilocus microsatellite typing reveals a genetic relationship but, also, genetic differences between Indian strains of Leishmania tropica causing cutaneous leishmaniasis and those causing visceral leishmaniasis. Parasites & vectors 7: 123.

9. Kumar NP, Srinivasan R, Anish T, Nandakumar G, Jambulingam P (2015) Cutaneous leishmaniasis caused by Leishmania donovani in the tribal population of the Agasthyamala Biosphere Reserve forest, Western Ghats, Kerala, India. Journal of medical microbiology 64: 157-163.

10. Sharma NL, Mahajan VK, Kanga A, Sood A, Katoch VM, et al. (2005) Localized cutaneous leishmaniasis due to Leishmania donovani and Leishmania tropica: preliminary findings of the study of 161 new cases from a new endemic focus in Himachal Pradesh, India. The American journal of tropical medicine and hygiene 72: 819-824.

11. Alborzi A, Pouladfar GR, Fakhar M, Motazedian MH, Hatam GR, et al. (2008) Isolation of Leishmania tropica from a patient with visceral leishmaniasis and disseminated cutaneous leishmaniasis, southern Iran. The American journal of tropical medicine and hygiene 79: 435-437.

12. Alborzi A, Rasouli M, Shamsizadeh A (2006) Leishmania tropica–isolated patient with visceral leishmaniasis in southern Iran. The American journal of tropical medicine and hygiene 74: 306-307.

13. Karamian M, Motazedian MH, Mehrabani D, Gholami K (2007) Leishmania major infection in a patient with visceral leishmaniasis: treatment with Amphotericin B. Parasitology research 101: 1431-1434.

14. Magill AJ, Grogl M, Gasser Jr RA, Sun W, Oster CN (1993) Visceral infection caused by Leishmania tropica in veterans of Operation Desert Storm. New England Journal of Medicine 328: 1383-1387.

15. Gramiccia M (2003) The identification and variability of the parasites causing leishmaniasis in HIV-positive patients in Italy. Annals of Tropical Medicine & Parasitology 97: 65-73.

16. Mebrahtu Y, Lawyer P, Githure J, Were JB, Muigai R, et al. (1989) Visceral leishmaniasis unresponsive to pentostam caused by Leishmania tropica in Kenya. The American journal of tropical medicine and hygiene 41: 289-294.

17. Mebrahtu YB, Van Eys G, Guizani I, Lawyer PG, Pamba H, et al. (1993) Human cutaneous leishmaniasis caused by Leishmania donovani sl in Kenya. Transactions of the Royal Society of Tropical Medicine and Hygiene 87: 598-601.

18. Knio K, Baydoun E, Tawk R, Nuwayri-Salti N (2000) Isoenzyme characterization of Leishmania isolates from Lebanon and Syria. The American journal of tropical medicine and hygiene 63: 43-47.

19. Aoun K, Bouratbine A (2014) Cutaneous leishmaniasis in North Africa: a review. Parasite 21.

20. Pun SB, Pandey K, Shah R (2013) A series of case reports of autochthonous visceral leishmaniasis, mostly in non-endemic hilly areas of Nepal. The American journal of tropical medicine and hygiene 88: 227-229.

21. Azmi K, Schonian G, Schnur LF, Nasereddin A, Ereqat S, et al. (2013) Development of assays using hexokinase and phosphoglucomutase gene sequences that distinguish strains of Leishmania tropica from different zymodemes and microsatellite clusters and their application to Palestinian foci of cutaneous leishmaniasis. PLoS Negl Trop Dis 7: e2464.

22. Lopes L, Vasconcelos P, Borges-Costa J, Soares-Almeida L, Campino L, et al. (2013) An atypical case of cutaneous leishmaniasis caused by Leishmania infantum in Portugal. Dermatology online journal 19.

23. Karunaweera ND (2009) Leishmania donovani causing cutaneous leishmaniasis in Sri Lanka: a wolf in sheep's clothing? Trends in parasitology 25: 458-463.

24. Ranasinghe S, Zhang W-W, Wickremasinghe R, Abeygunasekera P, Chandrasekharan V, et al. (2012) Leishmania donovani zymodeme MON-37 isolated from an autochthonous visceral leishmaniasis patient in Sri Lanka. Pathogens and global health 106: 421-424.

25. Rutherford C (1978) Cutaneous leishmaniasis in Southern Africa-a case report. South African Medical Journal 53: 716-718.

26. Elamin E, Guizani I, Guerbouj S, Gramiccia M, El Hassan A, et al. (2008) Identification of Leishmania donovani as a cause of cutaneous leishmaniasis in Sudan. Transactions of the Royal Society of Tropical Medicine and Hygiene 102: 54-57.

27. Lee JYY, Hsu MML, Wang CY, Ho JC (2009) Indigenous cutaneous leishmaniasis in Taiwan: three additional cases in southern Taiwan. International journal of dermatology 48: 441-443.

28. Wang JR, Lee ST, Juan WH, Chuang WL, Hung SI, et al. (2008) Indigenous leishmaniasis in Taiwan: report of a case. International journal of dermatology 47: 40-43.

29. Suankratay C (2014) Autochthonous Leishmaniasis: an emerging zoonosis in Thailand. J Infect Dis Antimicrob Agents 31: 1-8.

30. Kallel K, Haouas N, Pratlong F, Kaouech E, Belhadj S, et al. (2008) Cutaneous leishmaniasis caused by Leishmania infantum MON-24 in Tunisia: extension of the focus to the center of the country. Bulletin de la Societe de pathologie exotique (1990) 101: 29-31.

31. Khatri ML, Di Muccio T, Fiorentino E, Gramiccia M (2016) Ongoing outbreak of cutaneous leishmaniasis in northwestern Yemen: clinicoepidemiologic, geographic, and taxonomic study. International Journal of Dermatology 55: 1210-1218.

32. Khatri ML, Di Muccio T, Gramiccia M (2009) Cutaneous leishmaniasis in North-Western Yemen: A clinicoepidemiologic study and Leishmania species identification by polymerase chain reaction–restriction fragment length polymorphism analysis. Journal of the American Academy of Dermatology 61: e15-e21.

33. Barral A, Pedral-Sampaio D, Grimaldi JG, Momen H, McMahon-Pratt D, et al. (1991) Leishmaniasis in Bahia, Brazil: evidence that Leishmania amazonensis produces a wide spectrum of clinical disease. The American journal of tropical medicine and hygiene 44: 536-546.

34. Castro LS, Franca AdO, Ferreira EdC, Hans Filho G, Higa Júnior MG, et al. (2016) Leishmania infantum as a causative agent of cutaneous leishmaniasis in the state of Mato Grosso do Sul, Brazil. Revista do Instituto de Medicina Tropical de São Paulo 58.

35. Ramírez JD, Hernández C, León CM, Ayala MS, Flórez C, et al. (2016) Taxonomy, diversity, temporal and geographical distribution of cutaneous leishmaniasis in Colombia: a retrospective study. Scientific reports 6.

36. Zeledón R, Hidalgo H, Víquez A, Urbina A (1989) Atypical cutaneous leishmaniasis in a semiarid region of north-west Costa Rica. Transactions of the Royal Society of Tropical Medicine and Hygiene 83: 786.

37. Noyes H, Chance M, Ponce C, Ponce E, Maingon R (1997) Leishmania chagasi: genotypically similar parasites from Honduras cause both visceral and cutaneous leishmaniasis in humans. Experimental parasitology 85: 264-273.

38. Simon S, Nacher M, Carme B, Basurko C, Roger A, et al. (2017) Cutaneous leishmaniasis in French Guiana: revising epidemiology with PCR-RFLP. Tropical medicine and health 45: 5.

39. Belli A, Garcia D, Palacios X, Rodriguez B, Valle S, et al. (1999) Widespread atypical cutaneous Leishmaniasis caused by Leishmania (L.) Chagasi in Nicaragua. The American journal of tropical medicine and hygiene 61: 380-385.

40. Clarke CF, Bradley KK, Wright JH, Glowicz J (2013) Emergence of Autochthonous Cutaneous Leishmaniasis in Northeastern Texas and Southeastern Oklahoma. The American Journal of Tropical Medicine and Hygiene 88: 157-161.

41. De Lima H, Rodríguez N, Feliciangeli M, Barrios M, Sosa A, et al. (2009) Cutaneous leishmaniasis due to Leishmania chagasi/Le. infantum in an endemic area of Guarico State, Venezuela. Transactions of the Royal Society of Tropical Medicine and Hygiene 103: 721-726.
