# Supplementary material for: Atypical leishmaniasis: A global perspective with emphasis on the Indian subcontinent
Source: PLoS Negl Trop Dis. 2018 Sep 27;12(9):e0006659. doi: 10.1371/journal.pntd.0006659 (PMC6159859; doi:10.1371/journal.pntd.0006659)
Supplement: S2 Table — (DOCX) [file pntd.0006659.s002.docx]

**S2 Table: Geographical Distribution of Classical and Atypical Leishmaniasis with the causative agents in Indian Subcontinent**

| INDIA | | | | |
| --- | --- | --- | --- | --- |
| Disease type | **Causative agent** | **Prevalence** | **States** | **References** |
| Classical VL | ***L. donovani*** | **Endemic** | Bihar, West Bengal, Uttar Pradesh, Jharkhand, Uttarakhand*, Himachal Pradesh* | [[1-5](#_ENREF_1)] |
|  |  | **Resurgent cases** | Gujarat, Assam, Tamil Nadu | [[6](#_ENREF_6)] |
|  |  | **Sporadic cases** | Kerala, Madhya Pradesh, Haryana, Jammu & Kashmir* | [[7-11](#_ENREF_7)] |
| Atypical VL | ***L. tropica*** | **Few cases** | Himachal Pradesh, Bihar, West Bengal | [[12-16](#_ENREF_12)] |
| Classical CL | ***L. tropica* and *L. major*** | **Endemic** | Rajasthan | [[17-20](#_ENREF_17)] |
|  | ***L. tropica*** | **Endemic** | Jammu & Kashmir | [[21](#_ENREF_21),[22](#_ENREF_22)] |
|  |  | **Sporadic cases** | Punjab, Assam, Haryana, Delhi, Varanasi (Uttar Pradesh) | [[21](#_ENREF_21),[23](#_ENREF_23)] |
| Atypical CL | ***L. donovani*** | **Endemic*** | Kerala*, Himachal Pradesh* | [[15](#_ENREF_15),[16](#_ENREF_16),[24-30](#_ENREF_24)] |
| SRI LANKA | | | | |
| Disease type | **Causative agent** | **Prevalence** | **Districts** | **References** |
| Classical VL | ***L. donovani*** | **Few cases*** | Anuradhapura, Vavunia | [[31](#_ENREF_31)] |
| Atypical CL | ***L. donovani*** | **Endemic*** | Anuradhapura, Kurunegala, Matara, Hambantota, Polonnaruua, Monaragala | [[31](#_ENREF_31)-33] |
|  |  | **Few cases*** | Trincomalee, Vavunia, Matale, Galle, Puttalam, Kandy, Kegalle, Ratnapura, Kalutara, Ampara, Badulla, Batticaloa, Mallaitivu, Kilinochchi, Gampaha, Colombo | [[32](#_ENREF_32),3[4](#_ENREF_33)] |
|  | **NEPAL^#1^** | | |  |
| Disease type | **Causative agent** | **Prevalence** | **Districts** | **References** |
| Classical VL | ***L. donovani*** | **Endemic** | Parsa, Bara, Rautahat, Sarlahi, Mahottari, Dhanusha, Siraha, Saptari, Sunsari, Morang, Jhapa, Udaypur. | [3[5](#_ENREF_34)] |
|  |  | **Few cases*** | Doti, Bhojpur, Bajura, Dailekh, Surkhet, Pyuthan, NawalParsi, Sinduli, Okhaldhunga, Accham, Bardiya, | [[35-38](#_ENREF_34)] |
|  | **BHUTAN** | | |  |
| Disease type | **Causative agent** | **Prevalence** | **Districts** | **References** |
| Classical VL | ***L. donovani*** | **Few cases*** | Mongar, Trashiyangtse, Tsirang, Trashigang, Samtse, Lhuentse, Zhemgang | [3[9](#_ENREF_38)] |
|  | **BANGLADESH^#1^** | | |  |
| Disease type | **Causative agent** | **Prevalence** | **Districts** | **Reference** |
| Classical VL | ***L. donovani*** | **Endemic** | Rajshahi, Mymensingh, Rajbari, Jhenaidah, Patuakhali, Thakurgaon, Dinajpur, Nawabganj, Khulna, Magura, Panchaghar, Gaibanda, Joypurhat, Bogra, Narsingdi | [[3](#_ENREF_3),40 ,4[1](#_ENREF_40)] |
|  |  | **Few cases** | Barguna, Chandpur, Comillia, Dhaka, Faridpur, Gazipur, Gopalganj, Jamalpur, Jessore, Kishoreganj, Kushtia, Manikganj, Munshiganj, Naogaon, Natore, Narayanganj, Pabna, Rangpur, Sariatpur, Sirajganj, Tangail. | [40] |

**Endemic:** Regions with >25 annual case incidence (VL/CL) have been considered as endemic

**Few cases:** Regions with ≤ 25 annual case incidence (VL/CL) have been considered as having few cases

**Sporadic cases**: Occasional leishmaniasis cases occurring at irregular interval of times.

**Resurgent cases:** Re-emerging cases of leishmaniasis after eradication.

***** Newer disease sites discovered in recent years

**#1**VL elimination target (1 per 10,000 cases) accomplished.

**References:**

1. Ahmad S, Chandra H, Bhat NK, Dhar M, Shirazi N, et al. (2016) North Indian state of Uttarakhand: a new hothouse of visceral leishmaniasis. Tropical doctor 46: 111-113.

2. Kumar Bhat N, Ahuja V, Dhar M, Ahmad S, Pandita N, et al. (2017) Changing Epidemiology: A New Focus of Kala-azar at High-Altitude Garhwal Region of North India. Journal of tropical pediatrics 63: 104-108.

3. World Health Organization, 2010|2014, Leishmaniasis: Country Profiles (2016 July). Available from <http://www.who.int/leishmaniasis/burden/Country_profiles/en/>.

4. Chufal SS, Pant P, Chachra U, Singh P, Thapliyal N, et al. (2016) Role of Haematological Changes in Predicting Occurrence of Leishmaniasis-A Study in Kumaon Region of Uttarakhand. Journal of clinical and diagnostic research: JCDR 10: EC39.

5. Sharma NL, Mahajan VK, Ranjan N, Verma GK, Negi AK, et al. (2009) The sandflies of the Satluj river valley, Himachal Pradesh (India): some possible vectors of the parasite causing human cutaneous and visceral leishmaniases in this endemic focus. Journal of vector borne diseases 46: 136.

6. Dhiman RC, Pahwa S, Dhillon G, Dash AP (2010) Climate change and threat of vector-borne diseases in India: are we prepared? Parasitology Research 106: 763-773.

7. Bhat K, Pandita K, Khajuria A, Wani S (2014) Visceral leishmaniasis (kalazar) migrating West: A new autochthonous case from sub-Himalayas. Indian journal of medical microbiology 32: 94.

8. Dey A, Sharma U, Singh S (2007) First case of indigenous visceral leishmaniasis from central India. The American journal of tropical medicine and hygiene 77: 95-98.

9. Kaushal K, Veena M (2008) Two cases of Kala-azar in Haryana with no evidence of local transmission.

10. Nandedkar SS, Malukani K, Varma A (2011) Maiden visit of visceral leishmaniasis to Malwa region. The Journal of communicable diseases 43: 233-235.

11. Raina S, Raina RK, Sharma R, Rana BS, Bodh A, et al. (2016) Expansion of visceral leishmaniasis to northwest sub-Himalayan region of India: A case series. Journal of Vector Borne Diseases 53: 188.

12. Khanra S, Bandopadhyay SK, Chakraborty P, Datta S, Mondal D, et al. (2011) Characterization of the recent clinical isolates of Indian Kala-azar patients by RAPD-PCR method. Journal of Parasitic Diseases 35: 116-122.

13. Khanra S, Datta S, Mondal D, Saha P, Bandopadhyay SK, et al. (2012) RFLPs of ITS, ITS1 and hsp70 amplicons and sequencing of ITS1 of recent clinical isolates of Kala-azar from India and Bangladesh confirms the association of L. tropica with the disease. Acta tropica 124: 229-234.

14. Krayter L, Bumb RA, Azmi K, Wuttke J, Malik MD, et al. (2014) Multilocus microsatellite typing reveals a genetic relationship but, also, genetic differences between Indian strains of Leishmania tropica causing cutaneous leishmaniasis and those causing visceral leishmaniasis. Parasites & vectors 7: 123.

15. Sharma NL, Mahajan VK, Kanga A, Sood A, Katoch VM, et al. (2005) Localized cutaneous leishmaniasis due to Leishmania donovani and Leishmania tropica: preliminary findings of the study of 161 new cases from a new endemic focus in Himachal Pradesh, India. The American journal of tropical medicine and hygiene 72: 819-824.

16. Sharma NL, Mahajan VK, Negi AK, Verma GK (2009) The rK39 immunochromatic dipstick testing: a study for K39 seroprevalence in dogs and human leishmaniasis patients for possible animal reservoir of cutaneous and visceral leishmaniasis in endemic focus of Satluj river valley of Himachal Pradesh (India). Indian Journal of Dermatology, Venereology, and Leprology 75: 52.

17. Aara N, Khandelwal K, Bumb RA, Mehta RD, Ghiya BC, et al. (2013) Clinco-epidemiologic study of cutaneous leishmaniasis in Bikaner, Rajasthan, India. The American journal of tropical medicine and hygiene 89: 111-115.

18. Balai M, Gupta LK, Khare AK, Srivastava A, Mittal A, et al. (2016) Cutaneous leishmaniasis in a nonendemic area of South Rajasthan: A prospective study. Indian journal of dermatology 61: 521.

19. Dogra J, Aneja N, Lal BB, Mishra SN (1990) Cutaneous leishmaniasis in India. International journal of dermatology 29: 661-662.

20. Park K (2000) Park's Textbook of Preventive and Social Medicine. 16th edn.: M/S Banarsidas Bhanot Jabalpur, India. pp. 23-233.

21. Kaul N, Gupta V, Bhardwaj S, Dogra D, Dogra N (2016) A new focus of cutaneous leishmaniasis in Jammu division of Jammu and Kashmir State, India. Indian Journal of Dermatology, Venereology, and Leprology 82: 145.

22. Wani GM, Ahmad SM, Khursheed B (2014) Clinical study of cutaneous leishmaniasis in the Kashmir Valley. Indian dermatology online journal 6: 387-392.

23. Dhiman RC (2014) Emerging vector-borne zoonoses: eco-epidemiology and public health implications in India. Frontiers in public health 2.

24. Bora D, Khera A, Mittal V, Kaul S, Sharma R (1996) New focus of cutaneous leishmaniasis in India: Preliminary report. Indian Journal of Dermatology, Venereology, and Leprology 62: 19.

25. Kesavan A, Parvathy V, Thomas S, Sudha S (2003) Indigenous visceral leishmaniasis: two cases from Kerala. Indian pediatrics 40: 373-373.

26. Kumar NP, Srinivasan R, Anish T, Nandakumar G, Jambulingam P (2015) Cutaneous leishmaniasis caused by Leishmania donovani in the tribal population of the Agasthyamala Biosphere Reserve forest, Western Ghats, Kerala, India. Journal of medical microbiology 64: 157-163.

27. Nandha B, Srinivasan R, Jambulingam P (2014) Cutaneous leishmaniasis: knowledge, attitude and practices of the inhabitants of the Kani forest tribal settlements of Tiruvananthapuram district, Kerala, India. Health education research: cyu064.

28. Sahoo B, Kaur I, Radotra BD, Kumar B (2002) Cutaneous leishmaniasis in hilly areas of Himachal Pradesh (India). The Journal of dermatology 29: 248-249.

29. Sharma R, Mahajan V, Sharma N, Sharma A (2003) A new focus of cutaneous leishmaniasis in Himachal Pradesh (India). Indian Journal of Dermatology, Venereology, and Leprology 69: 170.

30. Simi S, Anish T, Jyothi R, Vijayakumar K, Rekha Rachel Philip NP (2010) Searching for cutaneous leishmaniasis in tribals from Kerala, India. Journal of global infectious diseases 2: 95.

31. Ranasinghe S, Zhang W-W, Wickremasinghe R, Abeygunasekera P, Chandrasekharan V, et al. (2012) Leishmania donovani zymodeme MON-37 isolated from an autochthonous visceral leishmaniasis patient in Sri Lanka. Pathogens and global health 106: 421-424.

32. Karunaweera ND (2009) Leishmania donovani causing cutaneous leishmaniasis in Sri Lanka: a wolf in sheep's clothing? Trends in parasitology 25: 458-463.

33. Weekly Epidemiological Report (2017) A publication of the Epidemiology Unit, Ministry of Health, Nutrition & Indigenous Medicine. <http://www.epid.gov.lk/web/images/pdf/wer/2017/vol_44_no_48-english.pdf>. Colombo, Srilanka.

34. World Health Organization (2011) Sri Lanka. <http://www.who.int/leishmaniasis/resources/SRI_LANKA.pdf>.

35. Pun SB, Pandey K, Shah R (2013) A series of case reports of autochthonous visceral leishmaniasis, mostly in non-endemic hilly areas of Nepal. The American journal of tropical medicine and hygiene 88: 227-229.

36. Ostyn B, Uranw S, Bhattarai NR, Das ML, Rai K, et al. (2015) Transmission of Leishmania donovani in the hills of Eastern Nepal, an outbreak investigation in Okhaldhunga and Bhojpur districts. PLoS Negl Trop Dis 9: e0003966.

37. Pandey BD, Pun SB, Kaneko O, Pandey K, Hirayama K (2011) Expansion of visceral leishmaniasis to the western hilly part of Nepal. The American journal of tropical medicine and hygiene 84: 107-108.

38. Schwarz D, Andrews J, Gauchan B (2011) Visceral leishmaniasis in far western Nepal: another case and concerns about a new area of endemicity. The American journal of tropical medicine and hygiene 84: 508-508.

39. Yangzom T, Cruz I, Bern C, Argaw D, den Boer M, et al. (2012) Endemic transmission of visceral leishmaniasis in Bhutan. The American journal of tropical medicine and hygiene 87: 1028-1037.

40. Ahmed B-N, Nabi SG, Rahman M, Selim S, Bashar A, et al. (2014) Kala-azar (visceral leishmaniasis) elimination in Bangladesh: successes and challenges. Current Tropical Medicine Reports 1: 163-169.

41. Huda MM, Chowdhury R, Ghosh D, Dash AP, Bhattacharya SK, et al. (2014) Visceral leishmaniasis-associated mortality in Bangladesh: a retrospective cross-sectional study. BMJ open 4: e005408.
